# Expression of activating transcription factor 5 (ATF5) is mediated by microRNA-520b-3p under diverse cellular stress in cancer cells

**DOI:** 10.1371/journal.pone.0225044

**Published:** 2020-06-30

**Authors:** Kari A. Gaither, Christy J. W. Watson, Bhanupriya Madarampalli, Philip Lazarus

**Affiliations:** 1 Department of Pharmaceutical Sciences, College of Pharmacy and Pharmaceutical Sciences, Washington State University, Spokane, WA, United States of America; 2 Department of Medicine, Rheumatology, University of Washington, Seattle, WA, United States of America; Universitat des Saarlandes, GERMANY

## Abstract

Cellular stress response mechanisms normally function to enhance survival and allow for cells to return to homeostasis following an adverse event. Cancer cells often co-opt these same mechanisms as a means to evade apoptosis and mitigate a state of constant cellular stress. Activating transcription factor 5 (ATF5) is upregulated under diverse stress conditions and is overexpressed in a variety of cancers. It was demonstrated ATF5 is a survival factor in transformed, but not normal cells. However, the regulation of ATF5 is not fully understood. The purpose of the present study was to investigate miRNA regulation at the 3’ untranslated region (UTR) of ATF5, with the goal of demonstrating a reversal of the upregulation of ATF5 induced under diverse cellular stress in cancer cells. A multifactorial approach using *in silico* analysis was employed to identify miRNAs 433-3p, 520b-3p, and 129-5p as potential regulators of ATF5, based on their predicted binding sites over the span of the ATF5 3’ UTR. Luciferase reporter assay data validated all three miRNA candidates by demonstrating direct binding to the target ATF5 3’. However, functional studies revealed miR-520b-3p as the sole candidate able to reverse the upregulation of ATF5 protein under diverse cellular stress. Additionally, miR-520b-3p levels were inversely related to ATF5 mRNA under endoplasmic reticulum stress and amino acid deprivation. This is the first evidence that regulation at the 3’ UTR is involved in modulating ATF5 levels under cellular stress and suggests an important role for miRNA-520b-3p in the regulation of ATF5.

## Introduction

Conserved defense mechanisms such as the heat shock response (HSR) and the adaptive integrated stress response (ISR) protect eukaryotic cells from environmental or physiological challenges to homeostasis [1, 2] Cancer cells face a hypoxic, nutrient poor environment, often with high levels of reactive oxygen species. Cancer cells are also characterized by unchecked growth, continuous proliferation, and increased DNA and protein synthesis, which leads to greater energy and nutrient requirements and a higher burden on the DNA repair systems and the protein assembly machinery. The HSR is the first line of defense triggered by proteotoxic stress stimulated by conditions in the tumor microenvironment and increased load on the protein folding machinery [3]. The HSR is frequently activated in cancer and high expression of chaperone heat shock proteins HSP70 and HSP27 is associated with poor prognosis in a variety of cancer types [3, 4]. An increase in protein production can additionally lead to endoplasmic reticulum (ER) stress since a portion of the proteins may require assembly in the ER, and cancer cells demonstrate an increased secretory nature, which further leads to a heavy ER load [5]. Moreover, ER stress signaling is altered in many cancer types and aids in tumor growth [5–7]. In an environment lacking in amino acids due to poor vascularization, and where continuous proliferation requires high amounts of protein production, amino acid limitation is a constant challenge. Cancer cells have adapted by shifting to the glycolytic pathway, which allows for the use of glycolysis intermediates for biosynthesis, and scavenging the microenvironment for nutrients; however, the metabolic needs of cancer cells are often unmet [8, 9].

This stress phenotype encompasses a variety of conditions, including hypoxia, oxidative stress, amino acid limitation, and endoplasmic reticulum (ER) stress [1, 10]. Cellular stress responses function to temporarily enhance cell survival and restore proper cellular function, or in times of prolonged or extreme cell stress, trigger cell death mechanisms. However, the ability of cells to co-opt the cell stress response to ensure survival can lead to an advantage in tumorigenesis. For example, cancer cells can become resistant to stress-induced cell death by developing a dependence on anti-apoptotic factors [11]. Additionally, enhanced cell survival can lead to epithelial to mesenchymal transition, and thus invasion and metastasis [12]. Apoptotic resistance can also contribute to treatment resistance towards many chemotherapeutic agents [10, 11].

In response to diverse stress conditions of the ISR (including ER stress and amino acid deprivation), stress activated protein kinases phosphorylate EIF2α, leading to a global reduction of protein translation. However, selective translation is initiated for some stress responsive proteins with multiple upstream open reading frames in the 5’ untranslated region (UTR). ATF5 is one such stress response protein. ATF5 is a transcription factor in the ATF/cAMP response-element binding protein (CREB) family and shares high homology to ATF4, but is less well characterized [13, 14]. The *Atf5* gene produces two distinct mRNA transcripts, ATF5α and ATF5β, differing only in the 5’ UTR and resulting in the same protein [15]. Both transcripts share high homology in humans and mice, and while ATF5α was found widely expressed in adult mice, the ATF5β was only detected during early development [15]. Like ATF4, ATF5α has two upstream open reading frames (ORFs), and under normal conditions, a re-initiation of translation occurs at a second upstream ORF, which overlaps the coding sequence ORF, inhibiting protein translation. However, under stress, the second upstream ORF is bypassed and post-transcriptional suppression is alleviated, a phenomenon observed to occur only with the ATF5α 5’ UTR but not the ATFβ 5’ UTR [16–18].

ATF5 is widely expressed and acts to regulate cell survival, cell cycle, homeostasis, and differentiation [14, 15, 19–23]. ATF5 protein expression is regulated at both the translational and post-translational level [16–18, 24, 25]. Under steady state conditions, ATF5 has a short half-life and is rapidly degraded due to proteasome and caspase dependent mechanisms [26]. However, ATF5 is elevated in response to cellular stress conditions such as amino-acid limitation, heat stress, ER stress, and oxidative stress and typically has a pro-survival effect via upregulation of its downstream targets, anti-apoptotic factors Mcl-1 and Bcl-2 [18, 27, 28]. Further, ATF5 is upregulated in a variety of carcinomas and is a survival factor in several cancer cell lines [20, 29]. When ATF5 function is blocked or silenced *in vitro*, apoptosis occurs in a number of cancer cells such as C6 glioma, SKOV-3 ovarian cancer, MCF-7, and other breast cancer cell lines, but not in non-transformed cells [20, 22, 29, 30]. Interference with ATF5 function *in vivo* causes cell death and tumor shrinkage of C6 glioma [29, 31]. It follows that ATF5 is a potential target for cancer therapy, and a better understanding of its regulation could lead to enhanced or novel therapeutics for cancer treatment.

It is well established that under stress conditions ATF5 is regulated at the post-transcriptional level via the 5’ UTR. Questions remain as to whether ATF5 is also regulated concurrently at the 3’ UTR by microRNA (miRNA). miRNA are evolutionarily conserved small non-coding RNAs ~22 nucleotides in length that regulate genetic expression at the translational level [32]. Briefly, a primary miRNA transcript is processed to the precursor miRNA (pre-miRNA) hairpin structure, which is then exported out of the nucleus and cleaved into a small double stranded RNA duplex, containing the mature single stranded miRNA [32]. The mature miRNA is loaded into the miRNA-induced silencing complex (miRISC) and recruits miRISC to the mRNA target site, resulting in repression of translation and/or mRNA degradation [32, 33].

Canonical miRNA seed sequences (nucleotides 2–7 at the 5’ end of the miRNA) bind via Watson Crick pairing to target sites in microRNA response elements (MRE) of mRNA, primarily located in the 3’ UTR [32, 34]. A single miRNA may target multiple mRNA, while conversely multiple miRNA may target a single mRNA [35, 36]. This lends to the inherent ability of miRNA to dynamically fine tune genetic expression. There is evidence that multiple miRNA can work in an additive or synergistic manner to regulate mRNA levels [34, 37]. Such cooperative miRNA modules are reported to have preferred binding regions with a distance between them of greater than 130 but less than 360 nucleotides apart [37]. Furthermore, miRNAs are more effective at repressing translation when bound at either end of the 3’ UTR, and shortening of the 3’ UTR results in enhanced suppression of protein synthesis by positioning microRNA closer to the stop codon [34, 38].

Genetic regulation by miRNA is highly context dependent, and is cell-type and condition specific [39]. Although the biogenesis and function of miRNA are tightly regulated at multiple steps, dysregulation of miRNA often occurs under conditions of cellular stress. Both miRNA activity and expression can be decreased by a variety of mechanisms, including altered levels of proteins involved in biogenesis or function, sequestration into stress granules, interference by RNA binding proteins, or transcriptional regulation [33, 40]. Genetic expression is thereby modified in a variety of cellular stress related diseases [33].

The regulation of specific miRNA via the cellular stress response has been shown to modulate stress response proteins and impact cell fate. ATF4 mediated repression of the miR-106b-25 cluster under ER stress resulted in upregulation of the pro-apoptotic factor BIM, while an increase in miR-30c-2-3p led to a repression of the pro-survival transcription factor XBP1 [40, 41]. Similarly, a protein kinase R-like endoplasmic reticulum kinase (PERK) dependent decrease in miR-424 during ER stress was shown to upregulate ATF6 via a release of translational repression [42]. Lu et al. (2017) noted an inverse relationship of miR-214 and ATF4 under oxidative stress and demonstrated suppression of ATF4 by miR-214 and a resulting increase in cell survival [43].

The goal of the present study was to investigate the potential translational suppression of ATF5 expression by multiple predicted miRNA regulators under various stress conditions. As ATF5 is a stress responsive transcription factor, this could have ramifications in the treatment of a number of stress-related diseases, particularly cancer and cancer therapy resistance. Evidence is presented demonstrating that miR-520b-3p may play an important role in the regulation of ATF5 under conditions of cellular stress.

## Materials and methods

### Reagents

The pGL3-control and pRL-TK Renilla plasmids were purchased from Promega (Madison, WI). DNA oligos were obtained from Integrated DNA Technologies (Coralville, IA). The lentiviral vector pSIREN-RetroQ-ZsGreen and retroviral vector pleGFP-C1 were purchased from Clontech Laboratories, Inc (Mountain View, CA), and pGIPZ-ATF5 shRNA lentiviral plasmids targeting the ATF5 3’ UTR or coding sequence were purchased from Dharmacon, Inc. (Lafayette, CO). Restriction enzymes Nhe*I*, Xba*I*, BamH*I*, and EcoR*I* were purchased from New England Biolabs, Inc. (Ipswich, MA). Heat-inactivated fetal bovine serum (FBS) was purchased from Atlanta Biologicals, while dialyzed heat-inactivated FBS was from GE Healthcare. Roswell Park Memorial Institute (RPMI) 1640 medium with L-glutamine; Dulbecco’s modified Eagle’s medium (DMEM) 4.5g/L glucose, L-glutamine, and pyruvate; and DMEM with 4.5 g/L glucose and sodium pyruvate and without glutamine, methionine, and cystine were purchased from Corning (Corning, NY). Polyethylenimine (PEI) was purchased from Polysciences, Inc (Warrington, PA) and thapsigargin was purchased from EMD Millipore (Burlington, MA). Rabbit polyclonal anti-ATF5 (#SAB4500895) and mouse monoclonal anti-β-tubulin (#T0198, clone D66) were purchased from Sigma Aldrich (St. Louis, MO), and rabbit polyclonal anti-GRP78 (#ab21685) from Abcam (Cambridge, MA). Goat anti-rabbit (#1706515) and goat anti-mouse (#1706516) secondary antibodies conjugated to horseradish peroxidase were purchased from Bio-Rad Laboratories (Hercules, CA).

### Cell lines, culture conditions and stress treatments

Human cervical adenocarcinoma (HeLa, ATCC CCL-2) and human breast adenocarcinoma MCF-7 (ATCC HTB-22) cell lines were purchased from the American Type Culture Collection (ATCC Manassas, VA). HeLa cells were cultured in DMEM medium and MCF-7 cells in RPMI medium, each supplemented with 10% FBS and hereafter referred to as complete medium. Cells were grown at 37°C in 5% CO_2_ under a humidified atmosphere up to ∼60–70% confluence for all experiments. All the cells tested negative for mycoplasma when tested with Hoechst staining using epifluorescence microscopy at 100x magnification. DNA transfection was performed using 1 μg DNA, unless otherwise specified, at a ratio of 1:3 DNA to PEI for 48 h. Heat stress was induced by incubating cells in a 42°C humidified incubator for 2 h. A stock solution of thapsigargin (ThG) in DMSO was diluted in complete medium and added to cells to induce ER stress. For amino acid deprivation experiments, cells were washed twice with phosphate buffered saline and treated with DMEM lacking glutamine, methionine, and cystine, and supplemented with 10% dialyzed fetal bovine serum (dFBS). Treatment details were established via a time course trial for each stress type, and treatments are specified in the Results section.

### miRNA binding site predictions

*In silico* analysis was performed to identify putative miRNA targets in the human ATF5 3’ UTR. TargetScan version 7.1 was used to predict miRNA-mRNA binding partners [36] and miRanda algorithms were used to identify the alignment between the miRNA and the corresponding MRE within the ATF5 3’ UTR [44]. Candidates were selected based on their predicted ability to undergo canonical binding of at least 7 nucleotides (nt) perfect complementary base pairing of the seed sequence to targets in the 3’ UTR of ATF5. Only conserved miRNA were included, with the position of putative binding along the 3’ UTR as well as predicted free energy upon binding taken into consideration [34, 36, 37]. Additionally, candidates were evaluated only where mature miRNA resulting from a single arm (5’ or 3’) of the precursor miRNA targeted the 3’ UTR. To include miRNA with binding sites at each end of the 3’ UTR and an analysis of potential concerted target suppression by miRNA with a preferred binding distance of 130–360 base pairs between binding sites, potential miRNA candidates were segregated based on target site locations in three segments: 3’ UTR nucleotides 1–281, 282–562, and 563–844 [37]. Potential miRNA candidates were cross-referenced with miRbase, the central repository for curated miRNA, to include only high confidence miRNA annotations in our analysis [45]. Candidates in each 3’ UTR segment that met the preferred binding distance listed above were then ranked by context++ score percentile and total context++ score, excluding any target sites with conserved branch length of zero.

### Plasmid constructs for luciferase and functional assays

To produce the human ATF5 3’ UTR-containing pGL3 plasmid, the pGL3 control vector was digested with Xba*I*, gel purified, and treated with calf intestinal phosphatase to prevent recircularization. The ATF5 3’ UTR was amplified from HeLa cell genomic DNA using primers that included Nhe*I* and Xba*I* restriction sites, with 5’-CGTAGCGCTAGCAAGGGCAGGGGTGTGGCTTCT-3’ as the sense primer (located at +1 - +21 relative to the translation stop codon), and 5’-TGCTTCTCTAGAAGACAAGATGCACAAGCCAGAGGAA-3’ as the antisense primer (located at +835 - +860 relative to the translation stop codon). The amplified segment was then digested with Nhe*I* and Xba*I* and the ATF5 3’ UTR product was ligated into the pGL3 control vector in the multiple cloning region downstream of the firefly luciferase reporter gene using its Xba*I* site, which is compatible with Nhe*I*. Orientation was confirmed by restriction fragment digestion and direct sequencing. Mutation of the seed sites for miRNA candidates was performed using the Quickchange XL Site Directed Mutagenesis kit (Agilent Technologies, Inc, Santa Clara, CA) following the manufacturer’s protocol; the primers used for mutagenesis are listed in Table 1.

**Table 1:** List of primers used.

Mutagenesis Primers	
ATF5 3’ UTR MRE1 S^a^	5’-GCTTATGCTTGTAATCCCAGGTGGTTGGGAGGCCAAGGCAGGAG-3’
ATF5 3’ UTR MRE1 AS	5’-CTCCTGCCTTGGCCTCCCAACCACCTGGGATTACAAGCATAAGC-3’
ATF5 3’ UTR MRE2 S	5’-CCTTCCCTCCTTTCTCGTCCAAAGGTGGAAATGTTTGGCCTTAGTCAATG-3’
ATF5 3’ UTR MRE2 AS	5’-CATTGACTAAGGCCAAACATTTCCACCTTTGGACGAGAAAGGAGGGAAGG-3’
ATF5 3’ UTR MRE3 S	5’-GGGACCCATATCCTACAGGCTTTTAGCAGGCTAGGTGACCTTGG-3’
ATF5 3’ UTR MRE3 AS	5’-CCAAGGTCACCTAGCCTGCTAAAAGCCTGTAGGATATGGGTCCC-3’
ATF5 3’ UTR MRE4 S	5’-GTACTGATTTTTTTGGGAGGTTATGAGGTTAAATAAAACGAAACATTTCCTCTGGCT-3’
ATF5 3’ UTR MRE4 AS	5’-AGCCAGAGGAAATGTTTCGTTTTATTTAACCTCATAACCTCCCAAAAAAATCAGTAC-3’
**qPCR Primers**	
ATF5 S	5’-GGGTGCAGTGGCTTATGC-3’
ATF5 AS	5’-GCCCAGGCTGGTATTGAC-3’
RPLP0 S	5’-GTGGAAGTGACATCGTCTTTA-3’
RPLP0 AS	5’-ATGGTGTTCTTGCCCATC-3’
miR-520b-3p S	5’-AAAGTGCTTCCTTTTAGAGGGAAAAA-3’
SNORD44 AS	5’-GCAAATGCTGACTGAACATGAA-3’
Oligo dT adapter	5’-GCATAGACCTGAATGGCGGTAAGGGTGTGGTAGGCGAGACATTTTTTTTTTTTTTTTTTTT-3’
miR Universal Reverse	5’-GCATAGACCTGAATGGCGGTA-3’

^a^ S, sense, AS, antisense.

Precursor miRNA (pre-miRNA) stem loop sequences were obtained from miRbase (miRbase.org) for each miRNA candidate and flanked with 100 bp of native sequence at each end. Primers were designed with BamH*I* and EcoR*I* restriction sites for cloning into the pSIREN RetroQ vector under the hU6 promoter. The primers used were as follows (sense and antisense, respectively): 5’-TTCGGGATCCTGCATCTTTCTTTTCGAGTCCA-3’ and 5’-TACGGAATTCTCCAGCCTGGGCAATAGAC-3’ for pre-miR-520b, 5’-TTCGGGATCCTCTGGAAGGCTCTCCTC-3’ and 5’-TACGGAATTCCCGCAACATCTCCCCTATC-3’ for pre-miR-433, and 5’-TTCGGGATCCACTCCCCTCCTCCCCCTAG-3’ and 5’-TACGGAATTCAAAGGAGAGCCAGGAGACCC-3’ for pre-miR-129. The amplified fragments were digested with BamH*I* and EcoR*I* and ligated into the pSIREN RetroQ vector using standard protocols.

### Dual luciferase reporter assay

Luciferase activity was measured using the Dual-Luciferase Reporter Assay System from Promega (Madison, WI, USA), following the protocol for manual luminometers. HeLa cells were seeded into 6-well plates and co-transfected 24 h later with 750 ng of pGL3 control vector containing the ATF5 3’ UTR, 15 ng of Renilla plasmid, and 750 ng of either scrambled control, pSIREN RetroQ vectors expressing precursor miRNA, or a positive control, as described below. A pGIPZ lentiviral vector containing shRNA targeting the ATF5 coding sequence served as scrambled control, and a pSIREN RetroQ vector containing shRNA for the firefly luciferase gene served as a positive control. Transfections were carried out in HeLa cells using PEI as described above. Cells were harvested at 48 h and luciferase activity was measured using the FB12/Sirius single tube luminometer from Berthold Detection Systems, performed in quadruplicate with Renilla luciferase activity as an internal standard.

### Preparation of cell lysates and immunoblotting

Cell extracts were prepared in radio-immunoprecipitation assay (RIPA) lysis buffer with 1X protease inhibitor cocktail (Sigma Aldrich). Lysates were centrifuged at 5000g for 15 min at 4°C to remove cell debris. Protein concentration was determined using a Bradford protein assay kit (Bio-Rad). Proteins were separated by SDS-polyacrylamide gel electrophoresis (10%) and transferred to a PVDF membrane (ThermoFisher Scientific, Waltham, MA). The membranes were probed with primary antibodies for ATF5 (1:1000) and/or GRP78 (1:500). Equal loading was verified with a β-tubulin antibody (1:2000). GRP78 expression levels served as stress markers for ER stress and amino acid deprivation. Blots were visualized via the Bio-Rad Chemidoc XRS system using horseradish peroxidase-coupled species-specific secondary antibodies and ECL Western blotting detection reagent (Bio-Rad) according to manufacturer’s instructions. Densitometry analysis was performed using Bio-Rad ImageLab software.

### Quantitative real-time PCR

Total RNA was extracted from cells using TRIzol (Invitrogen, Carlsbad, CA) and cDNA for mRNA analysis was synthesized using SuperScript III first Strand synthesis kit (Invitrogen), each according to the manufacturer’s instructions. miRNA cDNA was synthesized using qScript microRNA cDNA Synthesis Kit (Quanta Biosciences, Beverly, MA), which includes a polyadenylation step prior to reverse transcription and the use of an oligo-dT adapter primer during first-strand cDNA synthesis. Primers specific for ATF5 and ribosomal protein lateral stalk subunit P0 (RPLP0) were used for mRNA analysis; since the ATF5 primers were specific for the ATF5 3’ UTR, both the α and β ATF5 transcripts are detected in this analysis. Forward primers specific to miR-520b-3p and small nucleolar RNA, C/D Box 44 (SNORD44), and a universal reverse primer specific to a unique sequence at the 5’ end of the adapter primer were used for miRNA analysis. Primer sequences are listed in Table 1. Quantitative gene expression analysis was performed using BlazeTaq^™^ SYBR Green qPCR Mix with ROX (GeneCopoeia, Rockville, MD). Quantitative PCR reactions were performed in 10 μl reactions using the StepOnePlus real-time PCR (Applied Biosystems, Foster City, CA) in 96-well plates. Gene expression was compared with an endogenous, internal control (RPLP0 for mRNA or SNORD44 for miRNA) using the ΔΔCt method [46].

### Statistical analysis

Statistical analysis was performed using GraphPad Prism 7.00 (GraphPad Software, La Jolla, CA). Samples were analyzed using the student t-test with Welch’s correction for unequal variance, or one-way analysis of variance (ANOVA) as appropriate. One-way ANOVA was followed by Dunnett’s method of comparison post hoc test. The ANOVA trend test was also performed for qPCR analysis of mRNA after stress treatment, as appropriate. A p-value of ≤ 0.05 was considered significant. All experiments were performed at least three times in triplicate.

## Results

### *In silico* analysis predictions for miRNA candidates targeting the ATF5 3’ UTR

**Fig 1:**
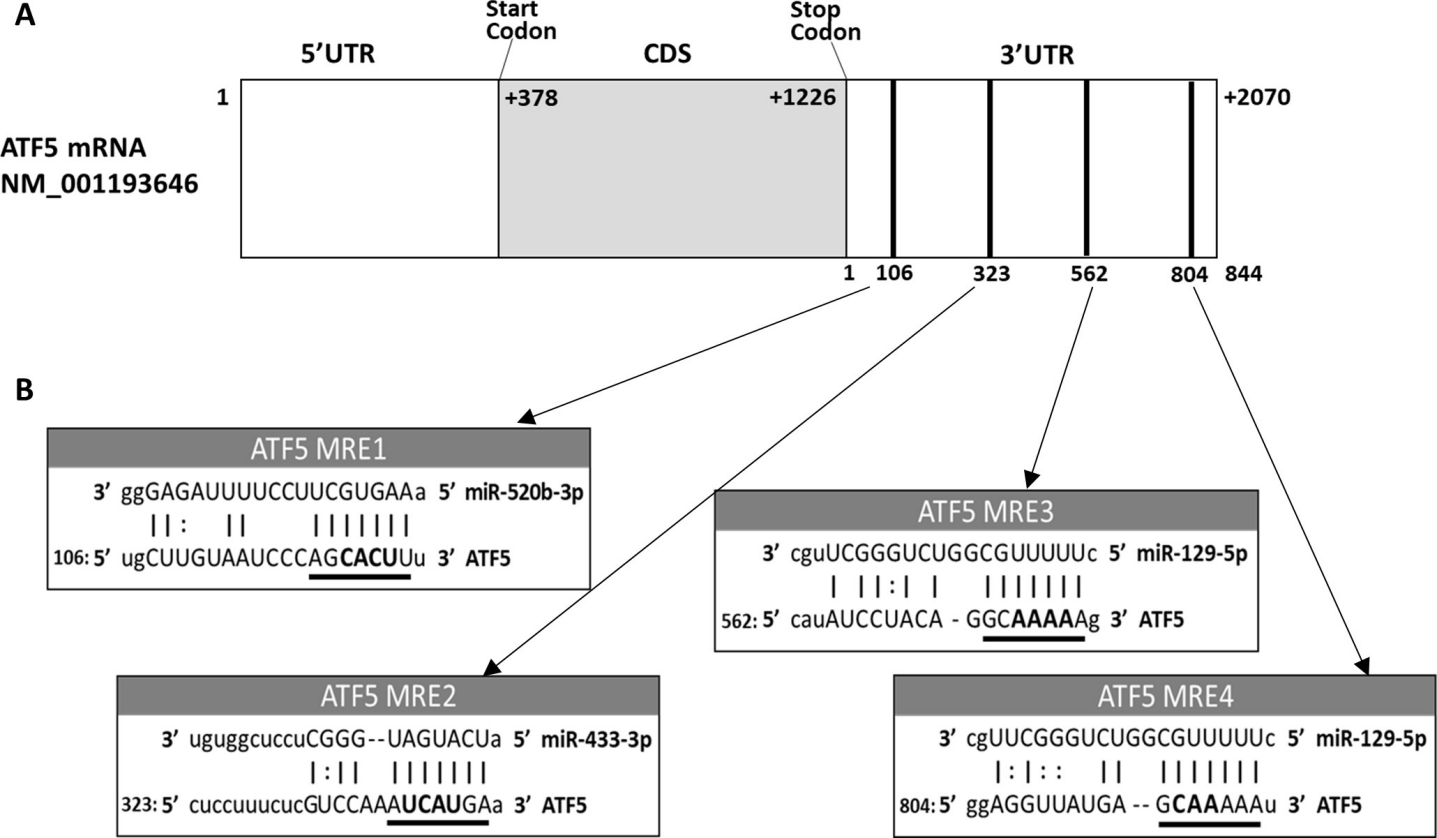
*In silico* predictions of potential miRNA candidates and their putative binding to the ATF5 3’ UTR. (A) Schematic of ATF5 mRNA. Nucleotides in the 3’ UTR are numbered from 1–844, with nt 1 indicating the 5’ end closest to the translation stop codon and nt 844 indicating the 3’ end closest to the polyA tail. Black bars in 3’ UTR depict binding sites of miRNA candidates along the 3’ UTR. (B) miRNA/ATF5 3’ UTR alignments. The ATF5 MREs of the selected miRNA candidates are referred to in order of distance from the ATF5 coding sequence. The miR-520b-3p MRE (ATF5 MRE1) is located at +106–126 nt and the miR-433-3p MRE (MRE2) is located at +323–346 nt. miR-129-5p has two MREs in the ATF5 3’ UTR, MRE3 and MRE4, located at +562–581 and + 804–522 nt, respectively. Bolded nucleotides in the underlined MRE seed sequences were mutated by site-directed mutagenesis to confirm specific binding of miRNA to the ATF5 3’ UTR in luciferase assays.

ATF5 is encoded by the gene *Atf5* on chromosome 19 and spans 5,235 bases (Entrez Gene ID: 22809). The ATF5α transcript is preferentially upregulated under cellular stress conditions and has a coding sequence of 849 nt (NCBI reference sequence NM_001193646) [16–18]. The 844 nt long 3’ UTR of ATF5 was analyzed by TargetScan to identify miRNA candidates that could potentially target sites within the ATF5 3’ UTR, with ATF5 3’ UTR divided into three segments for analysis. TargetScan predictions identified miR-520b-3p, miR-433-3p, and miR-129-5p as top miRNA candidates (Fig 1). In the segment closest to the coding sequence (nt 1–281), miR-520b was predicted to have a putative binding site within an MRE located at nt 106–125. miR-433-3p was predicted to have a binding site in the middle region (nt 282–562) within an MRE at nt 323–346. miR-129-5p had two predicted binding sites toward the 3’ end of the 3’ UTR within two MREs at nt 562–580 and 804–822.

### Predicted microRNA candidates target the ATF5 3’ UTR

**Fig 2:**
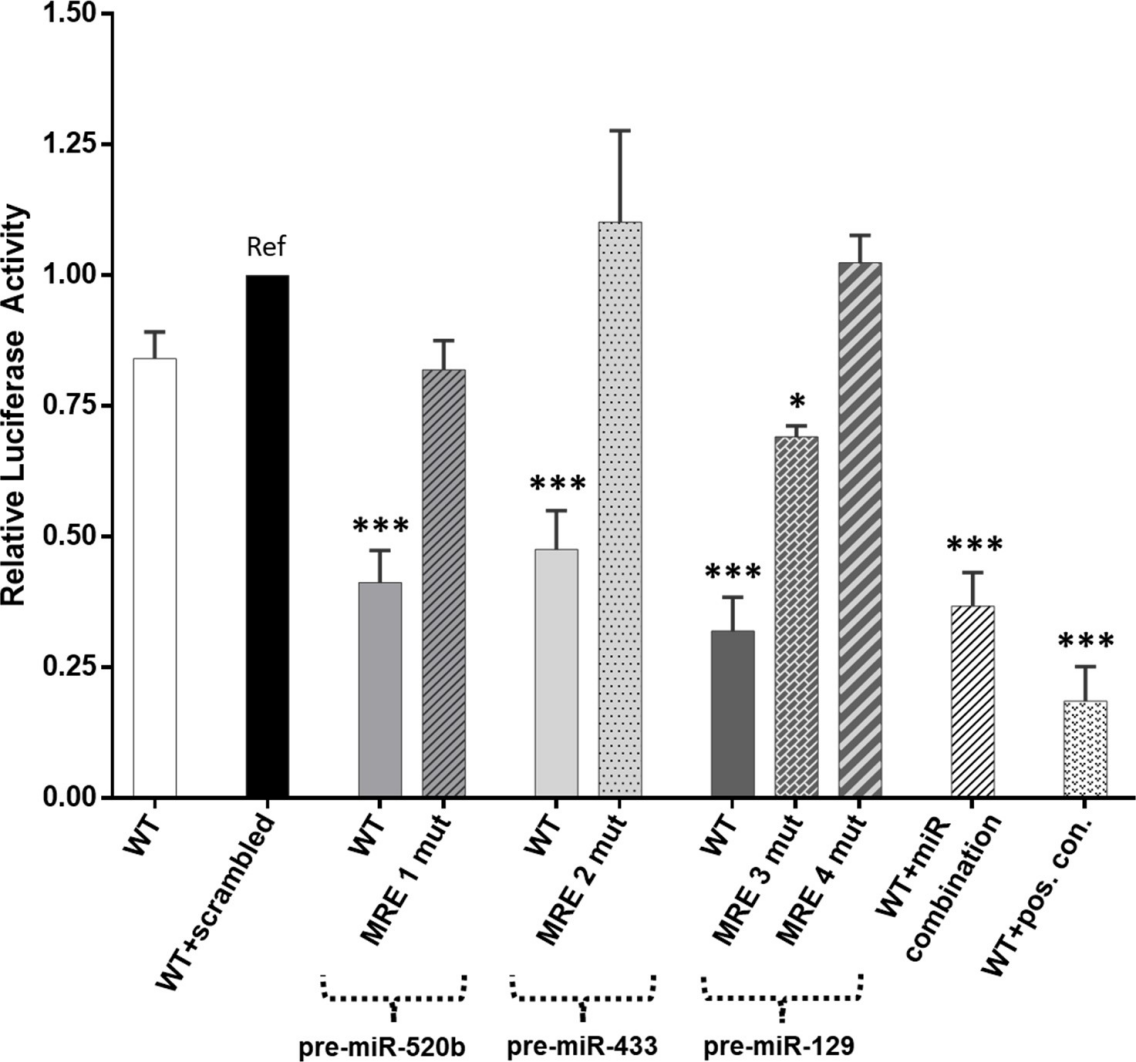
ATF5 3’ UTR luciferase activity in the presence of miRNA candidates. Luciferase activity for the wild-type ATF5 3’ UTR pGL3 vector alone or co-transfected with pre-miR-520b, pre-miR-433-3p, pre-miR-129-5p, a combination of all three, or a positive control (shRNA against luciferase). ATF5 3’ UTR MRE1-4 mutant pGL3 vectors (with deletions in the seed sequences) were co-transfected with pre-miRNA corresponding to the appropriate mature miRNA. All comparisons were made with wild-type ATF5 3’ UTR co-transfected with scrambled control. Columns represent the mean ± S.E. of at least three independent experiments performed in quadruplicate and normalized to the precursor miRNA scrambled control. * P<0.05, ** P<0.001. WT, wild-type.

Luciferase assays were used to confirm the predicted binding of miR-520b-3p, miR-433-3p, and miR-129-5p to the ATF5 3’ UTR. When the wild-type ATF5 3’ UTR-containing luciferase vector is co-transfected with either pre-miR-520b, pre-miR-433, or pre-miR-129, a significant (P<0.001) reduction in luciferase activity was observed (60%, 50%, and 70%, respectively), when compared to wild-type ATF5 3’ UTR co-transfected with a scrambled control (Fig 2). Co-transfection of the wild-type ATF5 3’ UTR with a combination of all three precursor miRNA candidates results in a significant (P<0.001) decrease of 60% in luciferase activity, similar to that observed for each of the three miRNA alone.

A 3–4 nt sequence in each MRE seed sequence (shown in Fig 1) was deleted within the ATF5 3’ UTR pGL3 plasmid and co-transfection was performed individually for each of these mutant plasmids and the corresponding precursor miRNA plasmid. Overexpression of MRE1, MRE2 and MRE4 mutants were able to restore luciferase activity to the approximate levels observed in the wild-type ATF5 3’ UTR-containing plasmid. However, luciferase activity remained significantly reduced when the MRE3 mutant was co-transfected with the pre-miR-129 plasmid.

### Experimental validation *in vitro* of miRNA-ATF5 3’ UTR binding predictions reveals activity of miR-520b-3p

**Fig 3:**
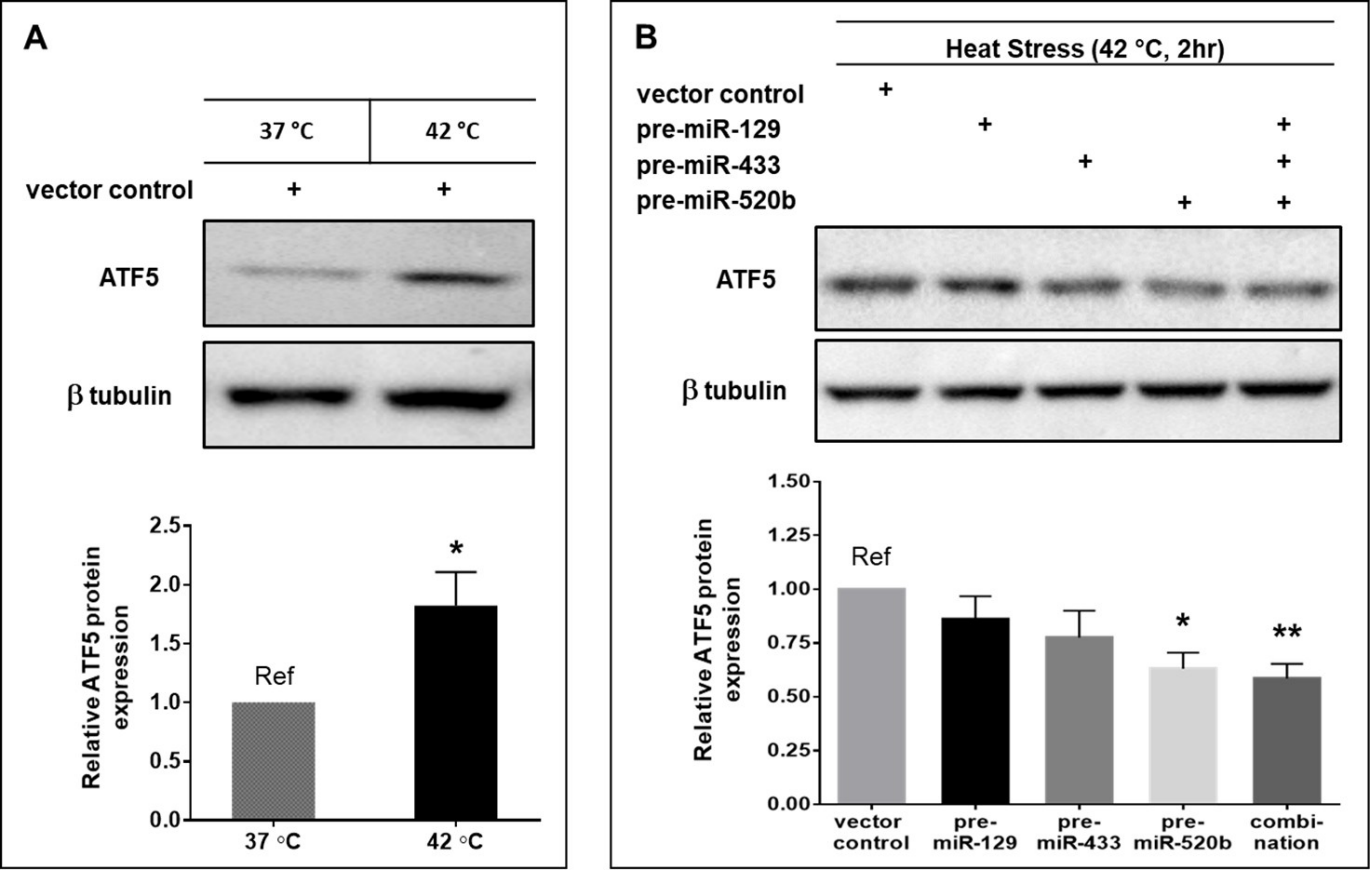
miR-520b-3p suppresses ATF5 protein expression under heat stress in HeLa cells. (A) HeLa cells were transfected with a vector control (pleGFP, 1 μg) and incubated at 37 or 42°C for 2 h. (B) HeLa cells were transfected with 1 μg of either vector control (pleGFP), pre-miR-129, pre-miR-433, or pre-miR-520b, or 0.5 μg each of all three precursor miRNA vectors (combination), and then subjected to 42°C for 2 h. Blots are representative images of 5 independent experiments. Columns represent the mean ± S.E. of ATF5 protein expression normalized to β-tubulin and compared to the reference (Ref). * P<0.05 and ** P<0.01.

Functional assays were performed to determine whether the miRNA candidates were able to regulate ATF5 expression at the protein level under diverse stress conditions. ATF5 protein is significantly upregulated in HeLa cells (45% increase, P<0.05) exposed to heat stress conditions of 42°C for 2 h (Fig 3A). miR-520b-3p was the only candidate able to significantly downregulate ATF5 protein (37% decrease, P<0.05) in HeLa cells under the same heat stress conditions (Fig 3B). Co-transfection of all three pre-miRNA vectors also resulted in a significant decrease in ATF5 protein expression (42% decrease, P<0.01), but did not appear to have an additive effect.

**Fig 4:**
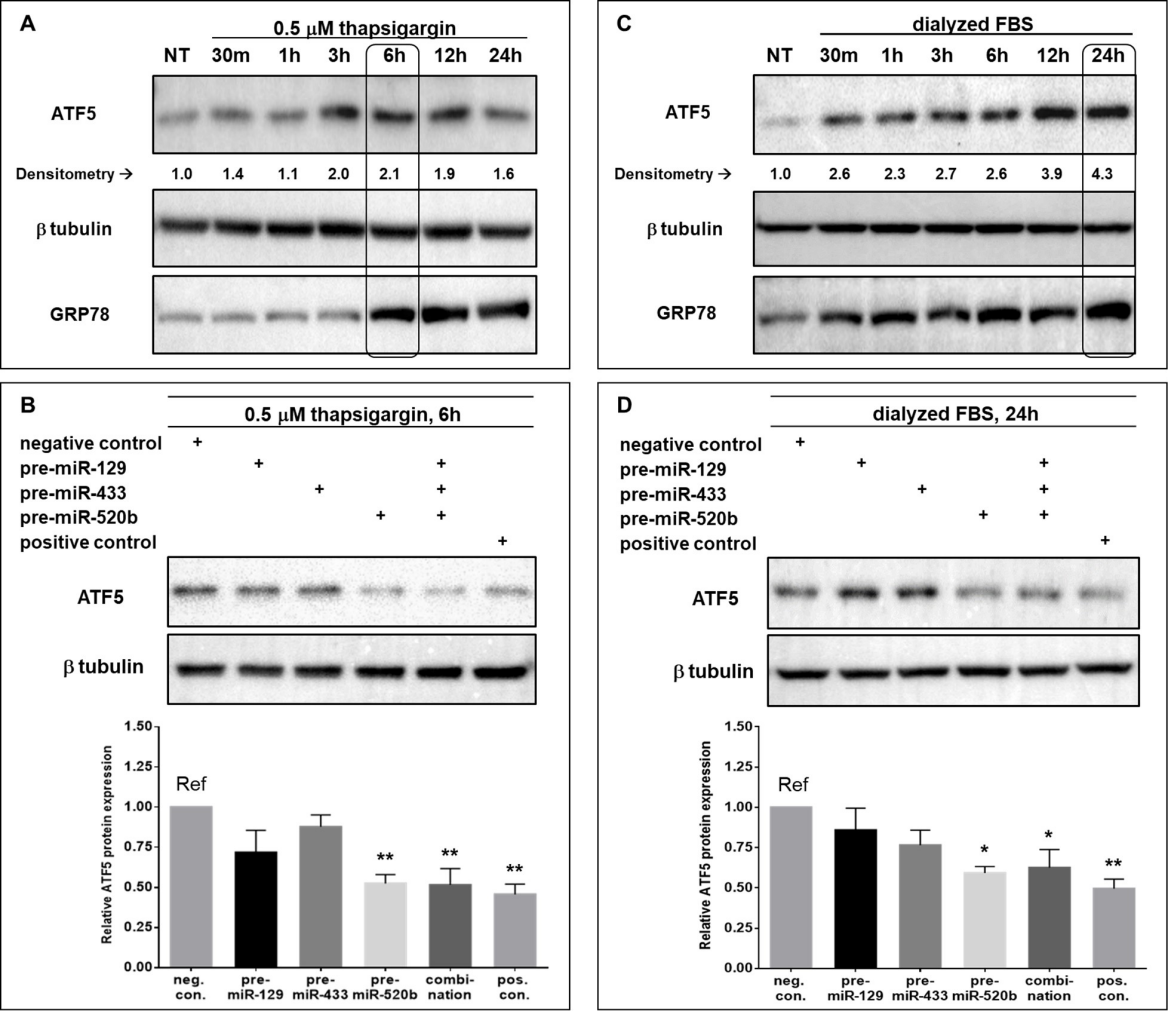
miR-520b-3p reverses ATF5 upregulation under ER Stress and amino acid deprivation in HeLa cells. **(**A) HeLa cells were transfected with 1 μg of sham vector (pleGFP) and treated with 0.5 μM ThG for the indicated times, with the exception of a non-treated control (NT). Membranes were probed for ATF5 protein expression, as well as GRP78 as an indicator of ER stress, normalized to β-tubulin and compared to NT. (B) HeLa cells were transfected with either 1 μg of negative control (shRNA against luciferase), pre-miR-129, pre-miR-433, pre-miR-520b, or positive control (shRNA against the ATF5 3’ UTR), or a combination of all three miRNAs (0.5 μg of each), and then treated with 0.5 μM ThG for 6 h prior to collection of cells. (C) HeLa cells were transfected as in panel A, and treated with DMEM lacking glutamine, methionine, and cystine supplemented with dFBS for the indicated times, with the exception of NT. Membranes were probed for ATF5 protein expression, as well as GRP78 as an indicator of amino acid deprivation-induced cellular stress, normalized to β-tubulin and compared to NT. (D) HeLa cells were transfected as in panel B and then treated with amino acid deprivation as in panel C for 24 h prior to collection of cells. Blots are representative images of 4 independent experiments. Columns represent the mean ± S.E. of ATF5 protein expression normalized to β-tubulin and compared to the reference (Ref). * P<0.05 and ** P<0.01.

We investigated the effect of miR-129-5p, miR-433-3p, and miR-520b-3p on ATF5 protein expression under the induction of two arms of the integrated stress response, ER stress and amino acid deprivation. Experimental conditions for ER stress and amino acid deprivation induction of ATF5 protein expression in HeLa cells were assessed via a time-course treatment of 0.5 μM ThG or media lacking glutamine, methionine, and cystine supplemented with dialyzed FBS, respectively. An upregulation of GRP78 protein expression indicates that 0.5 μM ThG is sufficient to induce ER stress after 6, 12, and 24 h of treatment, while ATF5 upregulation was pronounced at 3, 6, and 12 h (Fig 4A). Thus, using conditions of 0.5 μM ThG treatment for 6 h, we assessed the effect of each miRNA on ATF5 protein expression under ER stress via Western blot analysis. Compared to negative control, miRNA-520b-3p was the only candidate miRNA that significantly (P<0.01) downregulated ATF5 protein by 48% in HeLa cells under ER stress (Fig 4B). Co-transfection of all three pre-miRNA vectors resulted in a similar and significant (P<0.01) decrease of 48% in ATF5 protein expression. The observed decrease in ATF5 protein expression is comparable to that of the positive control (~54% decrease, P<0.01) (Fig 4B).

After treatment with glutamine-, methionine-, and cystine-free media supplemented with dFBS, GRP78 expression was upregulated as early as 30 min, but was most pronounced at 6, 12, and 24 h post-treatment, indicating amino acid deprivation-induced cellular stress in HeLa cells. Similarly, ATF5 protein levels were elevated as early as 30 min, but were markedly upregulated at 12 and 24 h post-treatment (Fig 4C). We assessed the effect of the candidate miRNA on ATF5 protein expression after 24 h of amino acid deprivation treatment via Western blot analysis. Compared to negative control, miRNA-520b-3p significantly (P<0.05) downregulated ATF5 protein by 41%. Co-transfection of all three pre-miRNA vectors also led to significantly (P<0.05) decreased ATF5 protein expression (38%). A comparable reduction of 50% in ATF5 protein expression was observed with the positive control-transfected cells (Fig 4D).

### Endogenous miR-520b-3p and ATF5 mRNA levels are altered in HeLa cells under cellular stress

**Fig 5:**
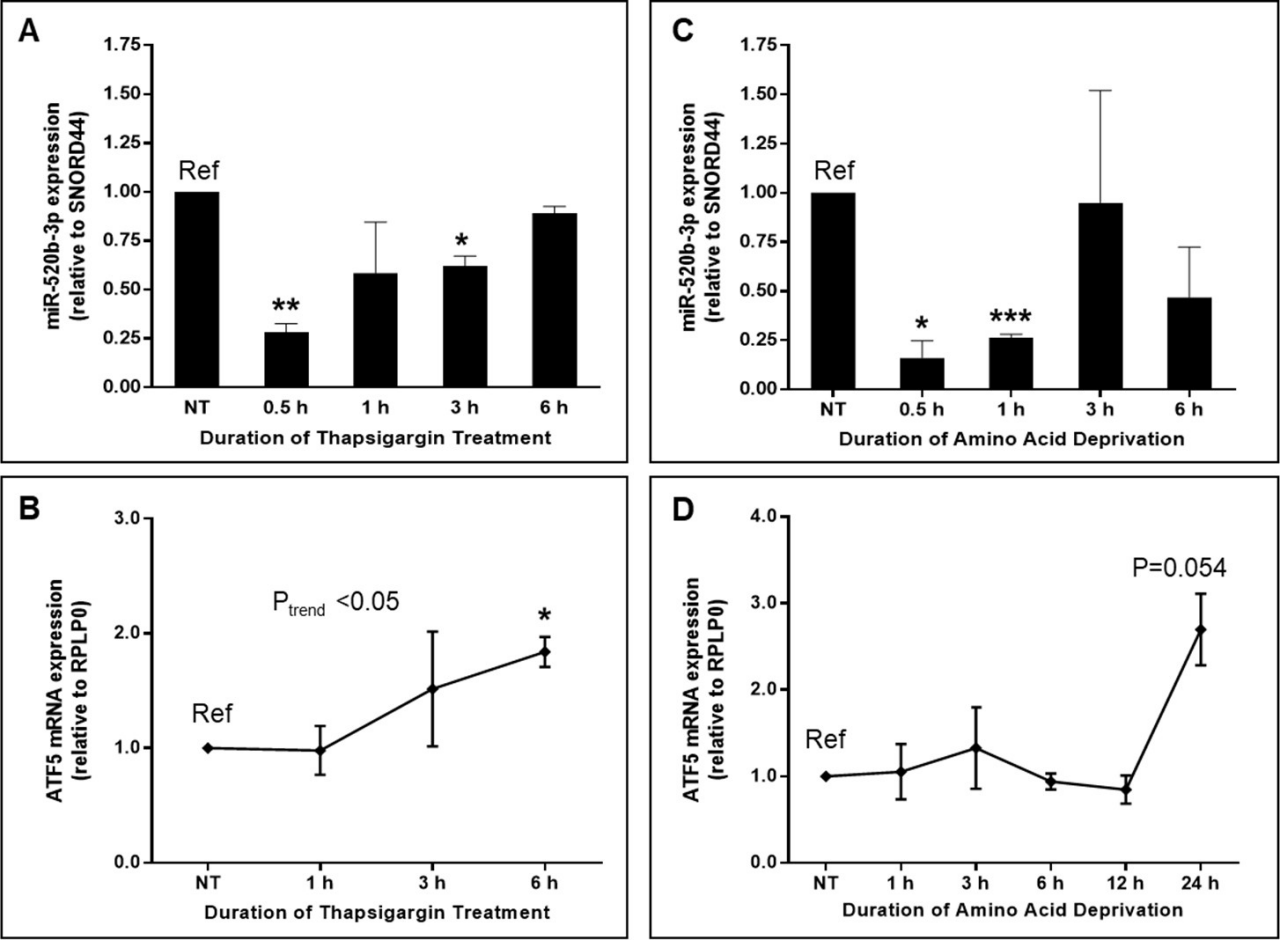
Diverse stress conditions trigger a rapid and dramatic decrease in miR-520b-3p and a corresponding increase in ATF5 mRNA over time in HeLa cells. (A) Endogenous miR-520b-3p expression levels were measured by qPCR in HeLa cells undergoing a time-course treatment of 0.5 μM ThG. (B) Matched samples were analyzed for ATF5 mRNA expression via qPCR. (C) Endogenous miR-520b-3p expression was assessed by qPCR in HeLa cells under amino acid deprivation. (D) ATF5 mRNA expression was measured in matched samples by qPCR. Expression levels are shown relative to non-treated control. miRNA expression was quantified against the SNORD44 endogenous control, while ATF5 mRNA expression was adjusted to the endogenous control gene RPLP0. Data points represent the mean ± S.E. of three independent replicates. * P<0.05, ** P<0.01, and *** P<0.001.

To further assess a functional effect of miR-520b-3p on ATF5 expression, we tested the impact of cellular stress on endogenous levels of miR-520b-3p and corresponding ATF5 mRNA expression. Cells underwent a time-course treatment of 0.5 μM ThG for ER stress or glutamine-, methionine-, and cystine-free media supplemented with dialyzed FBS for amino acid deprivation. Total RNA was extracted from cells at various time points and miR-520b-3p and ATF5 mRNA were measured via qPCR in matched samples. Under ER stress conditions, miR-520b-3p was significantly (P<0.01) downregulated as early as 0.5 h post-treatment (72% decrease compared to non-treated control), a trend that continued for up to 3h of treatment (Fig 5A). ATF5 mRNA was significantly (P<0.05) upregulated 1.8 fold at 6 h post-treatment (Fig 5B). A similar pattern was observed under conditions of amino acid deprivation, with miR-520b-3p expression significantly downregulated at both 0.5 h (84%) and 1 h (74%) post-treatment (P<0.05 and P<0.001, respectively; Fig 5C), while ATF5 mRNA was upregulated by 2.5-fold (P = 0.054) at 24 h post-treatment (Fig 5D).

### 520b-3p activity toward ATF5 3’ UTR is confirmed in the MCF-7 cell line

**Fig 6:**
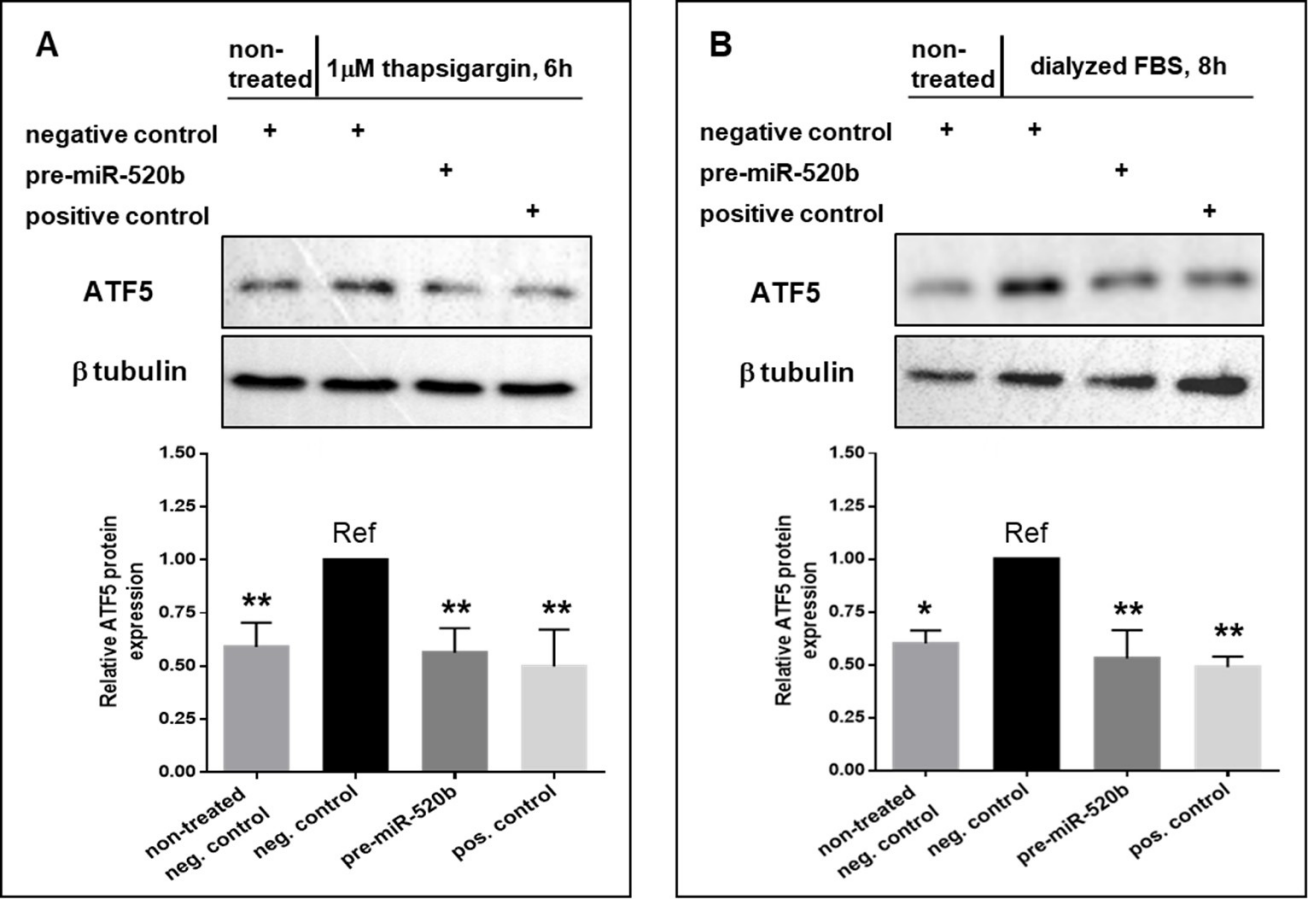
miR-520b-3p suppresses ATF5 upregulation under diverse stress conditions in MCF-7 cells. (A) MCF-7 cells were transfected with 1 μg of either negative control (shRNA against luciferase), pre-miR-520b, or a positive control (shRNA against ATF5 3’ UTR) and then treated with 1 μM ThG for 6 h prior to collection of cell lysates, with the exception of non-treated negative control. (B) MCF-7 cells were transfected as in (A) and treated with glutamine-, methionine-, and cystine-free media supplemented with dFBS for 8 h prior to collection of cell lysates, with the exception of non-treated negative control. Blots are representative images of 3 independent experiments. Columns represent the mean ± S.E. of ATF5 protein expression normalized to β-tubulin and compared the reference (Ref). * P<0.05 and ** P<0.01.

We further investigated the ability of miR-520b-3p to regulate ATF5 expression at the protein level in MCF-7 cells under similar experimental conditions. Using conditions of 1 μM ThG treatment for 6 h, we assessed the effect of miR-520b-3p on ATF5 protein expression in MCF-7 cells under ER stress. There was a 1.7-fold increase in ATF5 protein expression (P<0.01) in treated vs. non-treated MCF-7 cells transfected with the negative control luciferase shRNA (Fig 6A). In contrast, pre-miR-520b transfection in MCF-7 cells subsequently treated with ER stress resulted in a significant (P<0.01) 44% downregulation of ATF5 protein compared to negative control, a decrease similar to that observed for cells transfected with the ATF5 shRNA positive control (~44% decrease, P<0.01). We also evaluated the effect of miR-520b-3p on ATF5 protein expression in MCF-7 cells under amino acid deprivation conditions. Similar to that observed for ER stress conditions, ATF5 protein expression was significantly increased (1.8-fold, P<0.05) in treated cells as compared to non-treated cells, when each was transfected with the luciferase shRNA negative control (Fig 6B). In cells transfected with pre-miR-520b, ATF5 protein was again significantly downregulated compared to negative control cells (~47% decrease, P<0.05), a level of downregulation similar to that observed in the ATF5 shRNA positive control transfected cells (~51% decrease, P<0.01).

## Discussion

This study is the first to investigate the post-transcriptional regulation of ATF5 by miRNA in the context of cellular stress, and only the second to examine its regulation by miRNA overall. Here, the ability of multiple miRNA candidates to regulate the dynamic expression of ATF5, a stress-responsive transcription factor, was investigated under cellular stress conditions. Results from the present study show that miR-520b-3p is able to selectively bind the ATF5 3’ UTR *in vitro*, and its expression is inversely related to ATF5 mRNA levels in HeLa cells under both ER stress and amino acid deprivation. Moreover, miR-520b-3p can suppress endogenous ATF5 protein expression under diverse cellular stress conditions *in vitro*. This was seen in the case of heat stress, amino acid deprivation, and ER stress in HeLa cells, with the effects under amino acid deprivation and ER stress replicated in MCF-7 cells.

Post-transcriptional regulation of ATF5 by miR-520b-3p occurred in a significant and consistent manner, with a reduction in ATF5 protein of approximately 40% across all stress conditions in HeLa cells. Similarly, ATF5 was significantly decreased by 40–50% in MCF-7 cells transfected with precursor miR-520b under similar cellular stress conditions. These results were supported by a rapid and dramatic decrease in endogenous miR-520b-3p of greater than 70% as early as 0.5 h after treatment in HeLa cells under both ER stress and amino acid deprivation, which was inversely related to an increase in ATF5 mRNA over time of treatment in matched samples. Taken together, these findings suggest miR-520b-3p to be an important and novel contributor to the modulation of ATF5 expression under diverse stress conditions and across different cancer cell types. Recently, miR-141-3p was found to inhibit ATF5 protein synthesis and lead to a decrease in tumor size *in vivo* in glioma [47]. It would be useful to investigate miR-141-3p in the context of cellular stress, which was not examined in the previous study, as well as whether miR-520b-3p suppression of ATF5 expression could also lead to decreased tumor growth.

The ability of accurate prediction of miRNA-mRNA targeting is imperative to understanding miRNA regulation of genetic expression [36]. However, the extent to which miRNA act upon target mRNA translation and/or stability once bound is variable [34]. *In silico* algorithms attempt to gauge the potential of miRNA-mRNA binding and the expected efficacy of repression. However, Pinzon et al. (2017) cited a high rate of false positives by *in silico* modeling programs and a lack of reporting thereof [48]. It is important to note that the ability of miRNA to regulate expression of a gene is dependent on the context of the cell or tissue type and other conditions such as developmental or disease state [39]. Therefore, experimental confirmation of biological functions of miRNA is essential. The results presented in this study clearly illustrate this point. Luciferase assay data confirmed the ability of not only miR-520b-3p, but also miRNA candidates miR-433-3p and miR-129-5p, to interact with the ATF5 3’ UTR. Deletion of 3–4 nt sequences within the MRE sequences for the candidate miRNA reversed these effects, suggesting specificity of action, while confirming the binding of the mature miRNA candidates to the ATF5 3’ UTR in our system. However, *in vitro*, neither miR-433-3p nor miR-129-5p showed a significant effect on ATF5 protein levels. This is in contrast to the *in silico* data, which predicted miR-520b-3p to have the least efficacy for ATF5 post-transcriptional regulation out of the three candidates. The lack of effect from miR-129-5p was particularly surprising as it is known that microRNA with binding sites near either end of the 3’ UTR are more likely to affect protein translation and miRNA with multiple binding sites can have enhanced efficacy in target suppression [34]. As miR-129-5p did show a greater reduction in luciferase activity (about 20% over that of miR-433-3p), this may indeed account for some enhanced binding capacity. However, in the cellular context, there may be changes in mRNA 3D structure that do not allow efficient binding of miR-129-5p to the ATF5 3’ UTR, and which are not accounted for in our reporter system. Additionally, the RNA binding protein HUR, which binds AU-rich elements to stabilize mRNA in stress conditions, has been implicated in making target sites inaccessible for miRNA binding [49]. As the ATF5 3’ UTR has been reported to have at least one HUR binding site, it is possible that this mechanism could be negatively impacting the activity of miR-433-3p or miR-129-5p observed in the present study. Functional assays revealed that miR-520b-3p significantly suppressed endogenous ATF5 protein expression *in vitro* under varied cellular stress conditions. Hoffman et al. (2016) highlighted a greater potential impact of sites in the 3’ UTR closer to the stop codon and, as mentioned previously, it is known that miRNA post-transcriptional regulation is highly context specific [38, 39].

It is known that multiple miRNA can act in concert for an enhanced effect. Though it was expected that co-transfection of the three miRNA candidates would generate a cumulative suppression on ATF5 expression, this was not the case. The distance between miRNA binding sites has been investigated as an important factor in the ability of multiple miRNA to have a combined effect [34, 35]. A recent study determined that, overall, the majority of miRNA modules, or groups of miRNA that repressed a common mRNA target more than the individual miRNA, exhibited a preferred binding distance within one another of 130–360 nucleotides [37]. The average distance between binding sites for the three miRNA candidates is 213 nucleotides, which is well within this range. However, previous studies have shown preferred binding distances on the 3’ UTR to be less than 130 nucleotides [35]. This should be studied further for ATF5, as synergistic activity could enhance potential pharmacologic intervention.

Supporting the relevance of our findings that exogenously introduced miR-520b-3p reverses the upregulation of ATF5 protein expression in cancer cells under stress, we report a significant decrease in endogenous miR-520b-3p levels in HeLa cells exposed to both ER stress and amino acid deprivation. This likewise corresponds to a decrease in ATF5 mRNA over time of stress treatment. Although a lag time was observed in ATF5 mRNA upregulation as compared to the rapid miR-520b-3p decrease, this would be expected to some extent, as ATF5 is also post-transcriptionally regulated by upstream open reading frames in the 5’ UTR, which is known to impact ATF5 mRNA levels and is relieved upon stress-induced phosphorylation of eIF2α [16, 17]. Additionally, it is likely that miR-520-3p is acting primarily to inhibit translation, and the decrease of miR-520b-3p levels under cellular stress effectively primes the system, thus enabling the rapid increase in ATF5 protein translation seen upon stress induction. Further investigation into the mechanisms involved in the interplay between miRNA-520b modulation of ATF5 and its regulation at the 5’ UTR is warranted.

Evasion of apoptosis is one of the hallmarks of cancer [8]. ATF5 is a widely expressed transcription factor that is upregulated under diverse cellular stress conditions and typically leads to enhanced cell survival [18, 27, 28, 50]. With prolonged cellular stress, the adaptive stress response switches from pro-survival to activate apoptosis. However, cancer cells can become resistant to stress-induced apoptosis through upregulation of BCL-1 and MCL-2, both downstream targets of ATF5. Previous studies have found ATF5 is upregulated in a wide variety of cancers, and have linked ATF5 expression to a malignant phenotype, citing resistance to apoptosis, increased invasive capacity, and treatment resistance [20, 51–53]. Therefore, understanding the relationship between the upregulation of ATF5 due to cellular stress inherent to cancer cells and the effects of ATF5 on tumor progression and metastasis could have great therapeutic potential. Additionally, the use of cell stress to trigger apoptosis may enhance the function of various chemotherapeutic agents if the resistance to apoptosis can be overcome [54]. Thus, elucidating the mechanisms of ATF5 regulation under cellular stress could aid in the development of novel or enhanced cancer therapeutics. Further, miRNAs hold great promise for use as biomarkers for disease diagnosis and prognosis as well as potential targets or modes of treatment [55].

miRNA dysregulation leads to the impairment of fundamental cellular processes and is, therefore, associated with a number of different disease states, including cancer. While the mechanisms are not completely understood, both the expression and activity of miRNA can be altered. Potential scenarios include changes in the levels and activity of key proteins involved in miRNA biogenesis or function, miRNA sequestration into extracellular vesicles, and stress-dependent transcriptional changes in miRNA expression. Additionally, the RNA binding protein HUR has been shown to bind and sequester miRNA under cellular stress [49, 56]. It would be interesting to investigate further the specific mechanisms behind the suppression of miR-520b-3p seen under multiple stress conditions. Notably, miR-520b-3p is part of the miR-302-3p/372-3p/373-3p/520-3p miRNA family. Future studies could include assessing whether any members of this family may act as a redundant mechanism to aid in the suppression of ATF5 at the translational level.

In summary, a novel mechanism for modulation of ATF5 expression was identified in the present study. Specifically, the post-transcriptional regulation of ATF5 by miR-520b-3p, with a release of translational repression occurring via a reduction in miR-520b-3p levels upon exposure to diverse stress conditions is described. The findings presented in these studies are consistent with the role of miRNA acting as a buffer to reduce unwanted variation in protein levels under normal states, and to finely tune genetic expression in a rapid manner when conditions change, and with the potential role of ATF5 in stress-induced apoptosis and cancer progression. This study highlights a role for miR-520b-3p suppression in the regulation of ATF5 protein levels under cellular stress. The cellular stress response is adaptive and involves a complex network of systems. The data from the present study suggest an interplay between miR-520b-3p regulation of ATF5 expression at the 3’ UTR and the previously reported post-transcriptional suppression at the 5’ UTR. The extent to which these mechanisms cooperate to influence the dynamic regulation of ATF5 in stress and physiological disease states remains to be determined. Further studies are warranted to investigate the potential utility of miR-520b-3p as a therapeutic agent to re-sensitize cancer cells to chemotherapeutics as well as impact tumor growth via reversal of ATF5 expression in cancer cells.

## Supporting information

S1 Raw Images Full length Western blots for Figs 3, 4 and 6. (PDF)

## References

[1] Fulda S, Gorman AM, Hori O, Samali A. Cellular stress responses: cell survival and cell death. International journal of cell biology. 2010;2010:214074 10.1155/2010/214074 20182529 PMC2825543

[2] Pakos-Zebrucka K, Koryga I, Mnich K, Ljujic M, Samali A, Gorman AM. The integrated stress response. EMBO Rep. 2016;17(10):1374–95. 10.15252/embr.201642195 27629041 PMC5048378

[3] Dai C, Whitesell L, Rogers AB, Lindquist S. Heat shock factor 1 is a powerful multifaceted modifier of carcinogenesis. Cell. 2007;130(6):1005–18. 10.1016/j.cell.2007.07.020 17889646 PMC2586609

[4] Garrido C, Brunet M, Didelot C, Zermati Y, Schmitt E, Kroemer G. Heat shock proteins 27 and 70: anti-apoptotic proteins with tumorigenic properties. Cell Cycle. 2006;5(22):2592–601. 10.4161/cc.5.22.3448 17106261

[5] Dejeans N, Barroso K, Fernandez-Zapico ME, Samali A, Chevet E. Novel roles of the unfolded protein response in the control of tumor development and aggressiveness. Semin Cancer Biol. 2015;33:67–73. 10.1016/j.semcancer.2015.04.007 25953433

[6] Li J, Lee AS. Stress induction of GRP78/BiP and its role in cancer. Curr Mol Med. 2006;6(1):45–54. 10.2174/156652406775574523 16472112

[7] Chevet E, Hetz C, Samali A. Endoplasmic reticulum stress-activated cell reprogramming in oncogenesis. Cancer Discov. 2015;5(6):586–97. 10.1158/2159-8290.CD-14-1490 25977222

[8] Hanahan D, Weinberg RA. Hallmarks of cancer: the next generation. Cell. 2011;144(5):646–74. 10.1016/j.cell.2011.02.013 21376230

[9] Pavlova NN, Thompson CB. The Emerging Hallmarks of Cancer Metabolism. Cell Metab. 2016;23(1):27–47. 10.1016/j.cmet.2015.12.006 26771115 PMC4715268

[10] Nagel R, Semenova EA, Berns A. Drugging the addict: non-oncogene addiction as a target for cancer therapy. EMBO Rep. 2016;17(11):1516–31. 10.15252/embr.201643030 27702988 PMC5090709

[11] Certo M, Del Gaizo Moore V, Nishino M, Wei G, Korsmeyer S, Armstrong SA, et al Mitochondria primed by death signals determine cellular addiction to antiapoptotic BCL-2 family members. Cancer Cell. 2006;9(5):351–65. 10.1016/j.ccr.2006.03.027 16697956

[12] Shah PP, Beverly LJ. Regulation of VCP/p97 demonstrates the critical balance between cell death and epithelial-mesenchymal transition (EMT) downstream of ER stress. Oncotarget. 2015;6(19):17725–37. 10.18632/oncotarget.3918 25970786 PMC4627341

[13] Angelastro JM, Ignatova TN, Kukekov VG, Steindler DA, Stengren GB, Mendelsohn C, et al Regulated expression of ATF5 is required for the progression of neural progenitor cells to neurons. The Journal of neuroscience: the official journal of the Society for Neuroscience. 2003;23(11):4590–600. 12805299 10.1523/JNEUROSCI.23-11-04590.2003 PMC6740805

[14] Peters CS, Liang X, Li S, Kannan S, Peng Y, Taub R, et al ATF-7, a novel bZIP protein, interacts with the PRL-1 protein-tyrosine phosphatase. The Journal of biological chemistry. 2001;276(17):13718–26. 10.1074/jbc.M011562200 11278933

[15] Hansen MB, Mitchelmore C, Kjaerulff KM, Rasmussen TE, Pedersen KM, Jensen NA. Mouse Atf5: molecular cloning of two novel mRNAs, genomic organization, and odorant sensory neuron localization. Genomics. 2002;80(3):344–50. 10.1006/geno.2002.6838 12213205

[16] Hatano M, Umemura M, Kimura N, Yamazaki T, Takeda H, Nakano H, et al The 5'-untranslated region regulates ATF5 mRNA stability via nonsense-mediated mRNA decay in response to environmental stress. The FEBS journal. 2013;280(18):4693–707. 10.1111/febs.12440 23876217

[17] Watatani Y, Ichikawa K, Nakanishi N, Fujimoto M, Takeda H, Kimura N, et al Stress-induced translation of ATF5 mRNA is regulated by the 5'-untranslated region. The Journal of biological chemistry. 2008;283(5):2543–53. 10.1074/jbc.M707781200 18055463

[18] Zhou D, Palam LR, Jiang L, Narasimhan J, Staschke KA, Wek RC. Phosphorylation of eIF2 directs ATF5 translational control in response to diverse stress conditions. The Journal of biological chemistry. 2008;283(11):7064–73. 10.1074/jbc.M708530200 18195013

[19] Forgacs E, Gupta SK, Kerry JA, Semmes OJ. The bZIP transcription factor ATFx binds human T-cell leukemia virus type 1 (HTLV-1) Tax and represses HTLV-1 long terminal repeat-mediated transcription. Journal of virology. 2005;79(11):6932–9. 10.1128/JVI.79.11.6932-6939.2005 15890932 PMC1112100

[20] Monaco SE, Angelastro JM, Szabolcs M, Greene LA. The transcription factor ATF5 is widely expressed in carcinomas, and interference with its function selectively kills neoplastic, but not nontransformed, breast cell lines. International journal of cancer. 2007;120(9):1883–90. 10.1002/ijc.22469 17266024

[21] Pascual M, Gomez-Lechon MJ, Castell JV, Jover R. ATF5 is a highly abundant liver-enriched transcription factor that cooperates with constitutive androstane receptor in the transactivation of CYP2B6: implications in hepatic stress responses. Drug metabolism and disposition: the biological fate of chemicals. 2008;36(6):1063–72. 18332083 10.1124/dmd.107.019380

[22] Persengiev SP, Devireddy LR, Green MR. Inhibition of apoptosis by ATFx: a novel role for a member of the ATF/CREB family of mammalian bZIP transcription factors. Genes & development. 2002;16(14):1806–14. 12130540 10.1101/gad.992202 PMC186387

[23] Madarampalli B, Yuan Y, Liu D, Lengel K, Xu Y, Li G, et al ATF5 connects the pericentriolar materials to the proximal end of the mother centriole. Cell. 2015;162(3):580–92. 10.1016/j.cell.2015.06.055 26213385

[24] Pati D, Meistrich ML, Plon SE. Human Cdc34 and Rad6B ubiquitin-conjugating enzymes target repressors of cyclic AMP-induced transcription for proteolysis. Molecular and cellular biology. 1999;19(7):5001–13. 10.1128/mcb.19.7.5001 10373550 PMC84326

[25] Yuan Y, Gaither K, Kim E, Liu E, Hu M, Lengel K, et al SUMO2/3 modification of activating transcription factor 5 (ATF5) controls its dynamic translocation at the centrosome. The Journal of biological chemistry. 2018;293(8):2939–48. 10.1074/jbc.RA117.001151 29326161 PMC5827429

[26] Li G, Xu Y, Guan D, Liu Z, Liu DX. HSP70 protein promotes survival of C6 and U87 glioma cells by inhibition of ATF5 degradation. The Journal of biological chemistry. 2011;286(23):20251–9. 10.1074/jbc.M110.211771 21521685 PMC3121475

[27] Izumi S, Saito A, Kanemoto S, Kawasaki N, Asada R, Iwamoto H, et al The endoplasmic reticulum stress transducer BBF2H7 suppresses apoptosis by activating the ATF5-MCL1 pathway in growth plate cartilage. The Journal of biological chemistry. 2012;287(43):36190–200. 10.1074/jbc.M112.373746 22936798 PMC3476286

[28] Torres-Peraza JF, Engel T, Martin-Ibanez R, Sanz-Rodriguez A, Fernandez-Fernandez MR, Esgleas M, et al Protective neuronal induction of ATF5 in endoplasmic reticulum stress induced by status epilepticus. Brain: a journal of neurology. 2013;136(Pt 4):1161–76. 23518711 10.1093/brain/awt044

[29] Angelastro JM, Canoll PD, Kuo J, Weicker M, Costa A, Bruce JN, et al Selective destruction of glioblastoma cells by interference with the activity or expression of ATF5. Oncogene. 2006;25(6):907–16. 10.1038/sj.onc.1209116 16170340

[30] Chen A, Qian D, Wang B, Hu M, Lu J, Qi Y, et al ATF5 is overexpressed in epithelial ovarian carcinomas and interference with its function increases apoptosis through the downregulation of Bcl-2 in SKOV-3 cells. International journal of gynecological pathology: official journal of the International Society of Gynecological Pathologists. 2012;31(6):532–7. 23018213 10.1097/PGP.0b013e31824df26b

[31] Arias A, Lame MW, Santarelli L, Hen R, Greene LA, Angelastro JM. Regulated ATF5 loss-of-function in adult mice blocks formation and causes regression/eradication of gliomas. Oncogene. 2012;31(6):739–51. 10.1038/onc.2011.276 21725368 PMC3277917

[32] Bartel DP. MicroRNAs: target recognition and regulatory functions. Cell. 2009;136(2):215–33. 10.1016/j.cell.2009.01.002 19167326 PMC3794896

[33] Leung AK, Sharp PA. MicroRNA functions in stress responses. Molecular cell. 2010;40(2):205–15. 10.1016/j.molcel.2010.09.027 20965416 PMC2996264

[34] Grimson A, Farh KK, Johnston WK, Garrett-Engele P, Lim LP, Bartel DP. MicroRNA targeting specificity in mammals: determinants beyond seed pairing. Molecular cell. 2007;27(1):91–105. 10.1016/j.molcel.2007.06.017 17612493 PMC3800283

[35] Saetrom P, Heale BS, Snove O Jr., Aagaard L, Alluin J, Rossi JJ. Distance constraints between microRNA target sites dictate efficacy and cooperativity. Nucleic acids research. 2007;35(7):2333–42. 10.1093/nar/gkm133 17389647 PMC1874663

[36] Agarwal V, Bell GW, Nam JW, Bartel DP. Predicting effective microRNA target sites in mammalian mRNAs. Elife. 2015;4. 10.7554/eLife.05005 PMC4532895 26267216

[37] Ding J, Li X, Hu H. MicroRNA modules prefer to bind weak and unconventional target sites. Bioinformatics. 2015;31(9):1366–74. 10.1093/bioinformatics/btu833 25527098 PMC4410656

[38] Hoffman Y, Bublik DR, Ugalde AP, Elkon R, Biniashvili T, Agami R, et al 3' UTR shortening potentiates microRNA-based repression of pro-differentiation genes in proliferating human cells. PLoS Genet. 2016;12(2):e1005879 10.1371/journal.pgen.1005879 26908102 PMC4764332

[39] Erhard F, Haas J, Lieber D, Malterer G, Jaskiewicz L, Zavolan M, et al Widespread context dependency of microRNA-mediated regulation. Genome Res. 2014;24(6):906–19. 10.1101/gr.166702.113 24668909 PMC4032855

[40] Gupta S, Read DE, Deepti A, Cawley K, Gupta A, Oommen D, et al Perk-dependent repression of miR-106b-25 cluster is required for ER stress-induced apoptosis. Cell Death Dis. 2012;3:e333 10.1038/cddis.2012.74 22739985 PMC3388242

[41] Byrd AE, Aragon IV, Brewer JW. MicroRNA-30c-2* limits expression of proadaptive factor XBP1 in the unfolded protein response. J Cell Biol. 2012;196(6):689–98. 10.1083/jcb.201201077 22431749 PMC3308703

[42] Gupta A, Hossain MM, Read DE, Hetz C, Samali A, Gupta S. PERK regulated miR-424(322)-503 cluster fine-tunes activation of IRE1 and ATF6 during Unfolded Protein Response. Sci Rep. 2015;5:18304 10.1038/srep18304 26674075 PMC4682135

[43] Lu XZ, Yang ZH, Zhang HJ, Zhu LL, Mao XL, Yuan Y. MiR-214 protects MC3T3-E1 osteoblasts against H2O2-induced apoptosis by suppressing oxidative stress and targeting ATF4. Eur Rev Med Pharmaco. 2017;21(21):4762–70. 29164589

[44] Betel D, Koppal A, Agius P, Sander C, Leslie C. Comprehensive modeling of microRNA targets predicts functional non-conserved and non-canonical sites. Genome Biol. 2010;11(8):R90 10.1186/gb-2010-11-8-r90 20799968 PMC2945792

[45] Kozomara A, Griffiths-Jones S. miRBase: annotating high confidence microRNAs using deep sequencing data. Nucleic acids research. 2014;42(Database issue):D68–73. 10.1093/nar/gkt1181 24275495 PMC3965103

[46] Livak KJ, Schmittgen TD. Analysis of relative gene expression data using real-time quantitative PCR and the 2(-Delta Delta C(T)) Method. Methods. 2001;25(4):402–8. 11846609 10.1006/meth.2001.1262

[47] Wang M, Hu M, Li Z, Qian D, Wang B, Liu DX. miR-141-3p functions as a tumor suppressor modulating activating transcription factor 5 in glioma. Biochemical and biophysical research communications. 2017;490(4):1260–7. 10.1016/j.bbrc.2017.05.179 28595907 PMC5759330

[48] Pinzon N, Li B, Martinez L, Sergeeva A, Presumey J, Apparailly F, et al microRNA target prediction programs predict many false positives. Genome Res. 2017;27(2):234–45. 10.1101/gr.205146.116 28148562 PMC5287229

[49] Ahuja D, Goyal A, Ray PS. Interplay between RNA-binding protein HuR and microRNA-125b regulates p53 mRNA translation in response to genotoxic stress. RNA Biol. 2016;13(11):1152–65. 10.1080/15476286.2016.1229734 27592685 PMC5100343

[50] Watatani Y, Kimura N, Shimizu YI, Akiyama I, Tonaki D, Hirose H, et al Amino acid limitation induces expression of ATF5 mRNA at the post-transcriptional level. Life sciences. 2007;80(9):879–85. 10.1016/j.lfs.2006.11.013 17140605

[51] Ishihara S, Yasuda M, Ishizu A, Ishikawa M, Shirato H, Haga H. Activating transcription factor 5 enhances radioresistance and malignancy in cancer cells. Oncotarget. 2015;6(7):4602–14. 10.18632/oncotarget.2912 25682872 PMC4467102

[52] Karpel-Massler G, Horst BA, Shu C, Chau L, Tsujiuchi T, Bruce JN, et al A synthetic cell-penetrating dominant-negative ATF5 peptide exerts anticancer activity against a broad spectrum of treatment-resistant cancers. Clin Cancer Res. 2016;22(18):4698–711. 10.1158/1078-0432.CCR-15-2827 27126996 PMC5026557

[53] Nukuda A, Endoh H, Yasuda M, Mizutani T, Kawabata K, Haga H. Role of ATF5 in the invasive potential of diverse human cancer cell lines. Biochemical and biophysical research communications. 2016;474(3):509–14. 10.1016/j.bbrc.2016.04.131 27125458

[54] Avril T, Vauleon E, Chevet E. Endoplasmic reticulum stress signaling and chemotherapy resistance in solid cancers. Oncogenesis. 2017;6(8):e373 10.1038/oncsis.2017.72 28846078 PMC5608920

[55] Hayes J, Peruzzi PP, Lawler S. MicroRNAs in cancer: biomarkers, functions and therapy. Trends Mol Med. 2014;20(8):460–9. 10.1016/j.molmed.2014.06.005 25027972

[56] Poria DK, Guha A, Nandi I, Ray PS. RNA-binding protein HuR sequesters microRNA-21 to prevent translation repression of proinflammatory tumor suppressor gene programmed cell death 4. Oncogene. 2016;35(13):1703–15. 10.1038/onc.2015.235 26189797 PMC4820683

