# Supplementary material for: Expression of activating transcription factor 5 (ATF5) is mediated by microRNA-520b-3p under diverse cellular stress in cancer cells
Source: PLoS One. 2020 Jun 30;15(6):e0225044. doi: 10.1371/journal.pone.0225044 (PMC7326155; doi:10.1371/journal.pone.0225044)
Supplement: S1 Raw Images — (PDF) [file pone.0225044.s001.pdf]

Uncut western blot images from Figure 3.

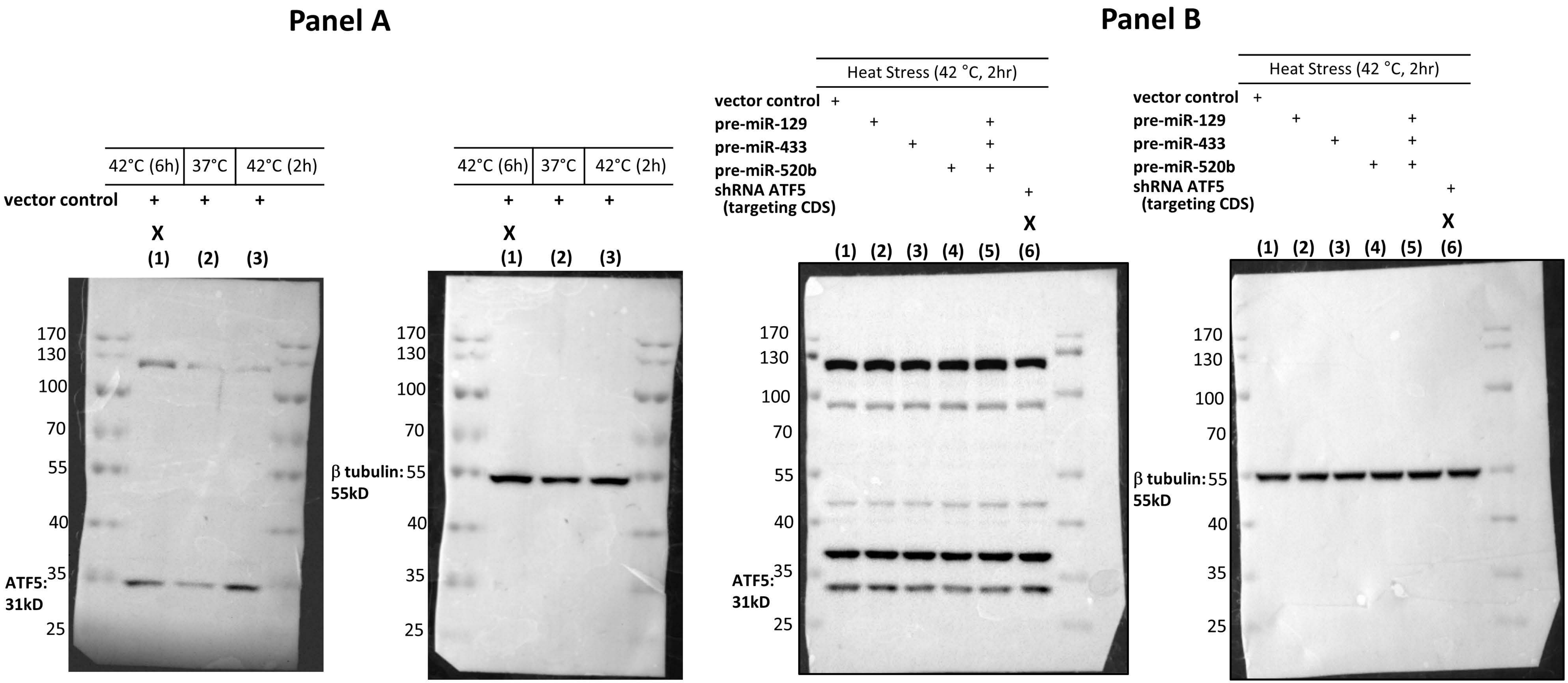

HeLa cells were transfected as described in manuscript and treated as indicated. ATF5, with a molecular weight ~ 31kD was probed, as was β tubulin with a molecular weight ~ 55kD. Images were captured using the Bio-Rad Chemidoc XRS, after incubation with horseradish peroxidase-coupled species-specific secondary antibodies and ECL Western blot detection reagent.

Uncut western blot images from Figure 4.

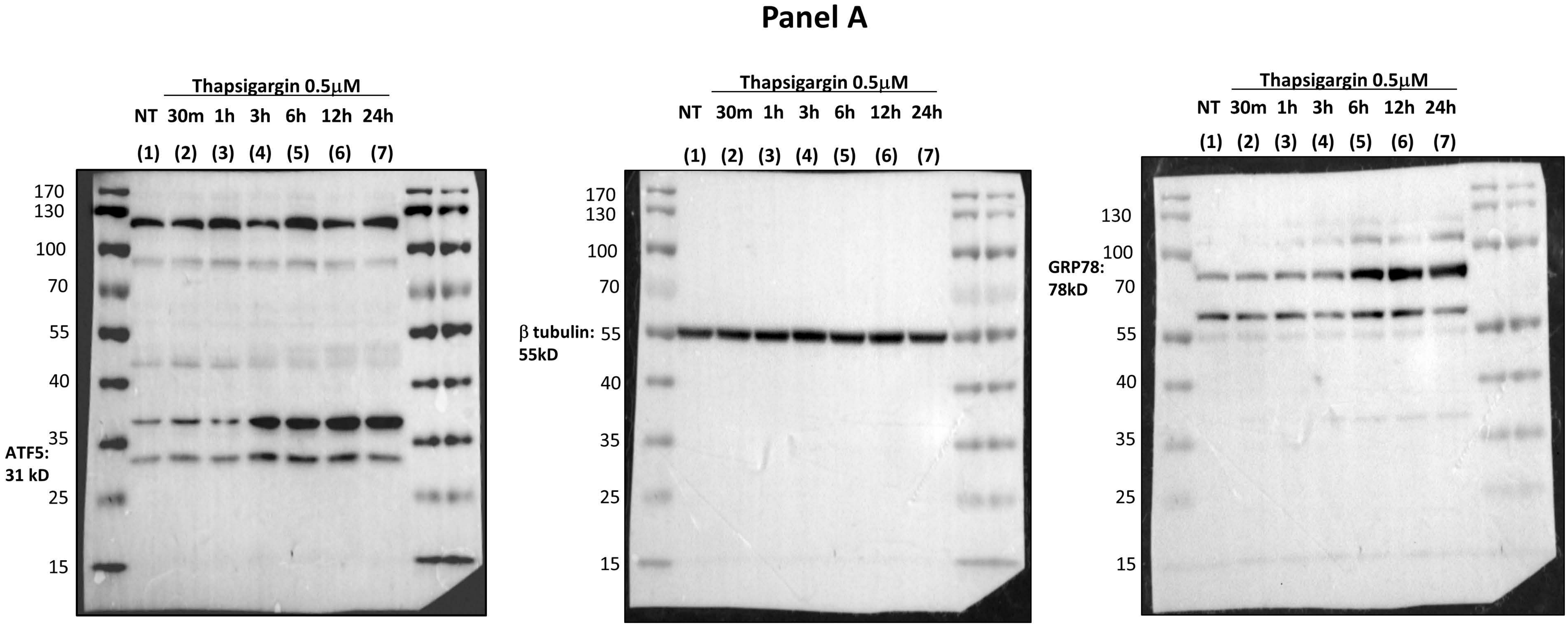

HeLa cells were transfected with vector control (GFP) and treated as described. NT= not treated. Blots were probed for ATF5, molecular weight ~31kD; β tubulin, molecular weight ~55kD, and GRP78, molecular weight ~78kD. Images were captured using the Bio-Rad Chemidoc XRS, after incubation with horseradish peroxidase-coupled species-specific secondary antibodies and ECL Western blot detection reagent.

Uncut western blot images from Figure 4.

Panel B

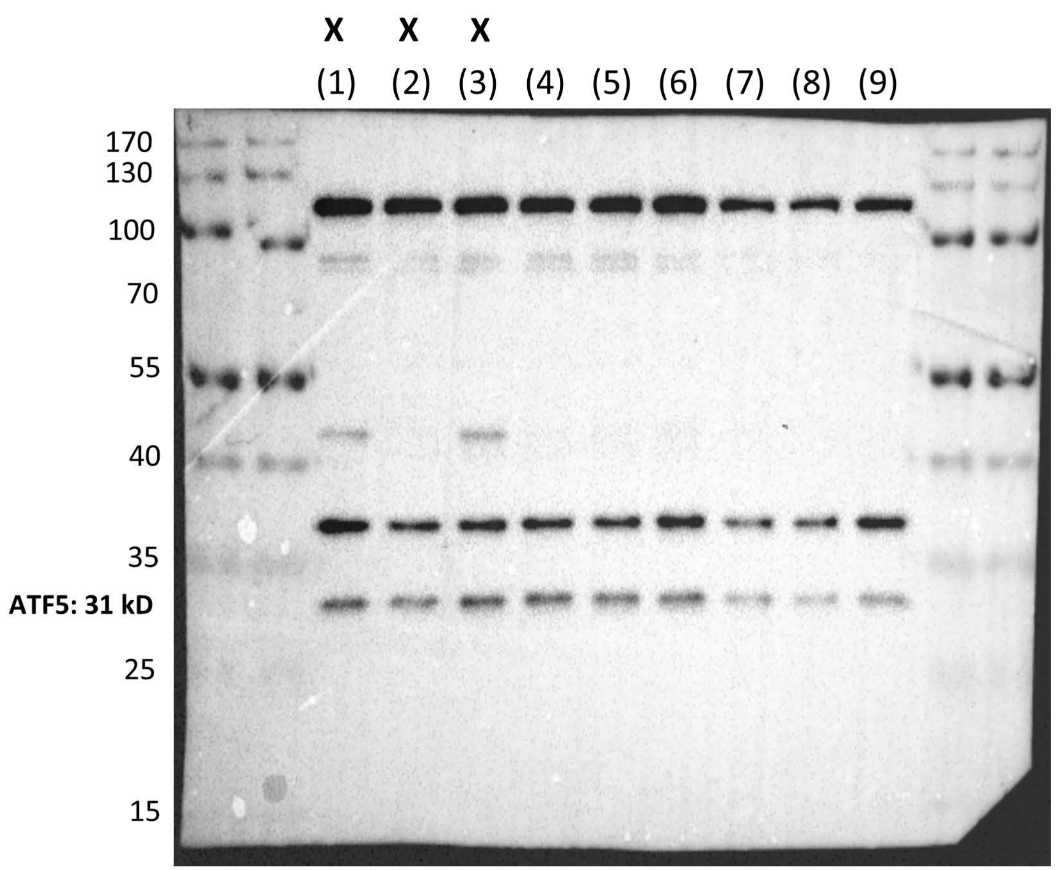

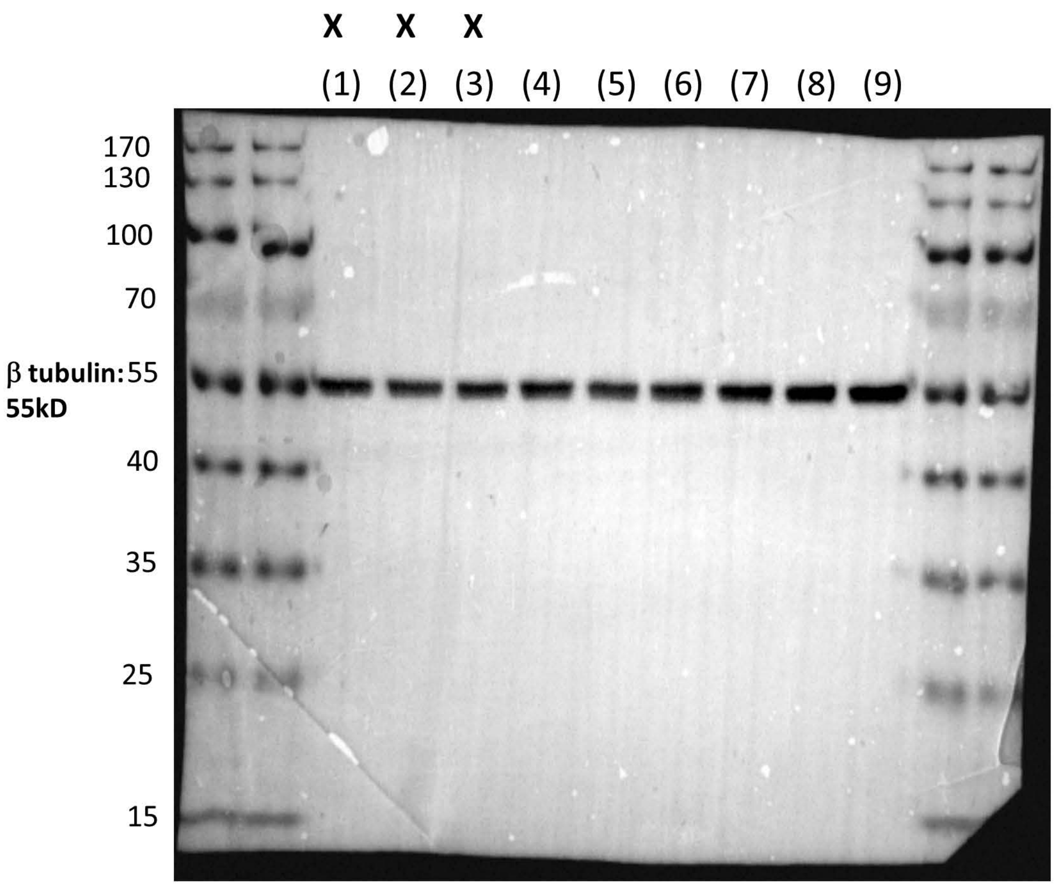

HeLa cells were transfected and treated with 0.5μM thapsigargin for 6h as described. Lanes 1-2: non-transfected, lane 3: neg. control, lane 4: neg. control, lane5: pre-miR-129, lane 6:, pre-miR-433-3p, lane 7: pre-miR-520b, lane 8: all three pre-miRNA co-transfected, lane 9: pos. control. ATF5, molecular weight ~31kD was probed, as was β tubulin, molecular weight ~55kD. Images were captured using the Bio-Rad Chemidoc XRS, after incubation with horseradish peroxidase-coupled species-specific secondary antibodies and ECL Western blot detection reagent.

Uncut western blot images from Figure 4.

Panel C

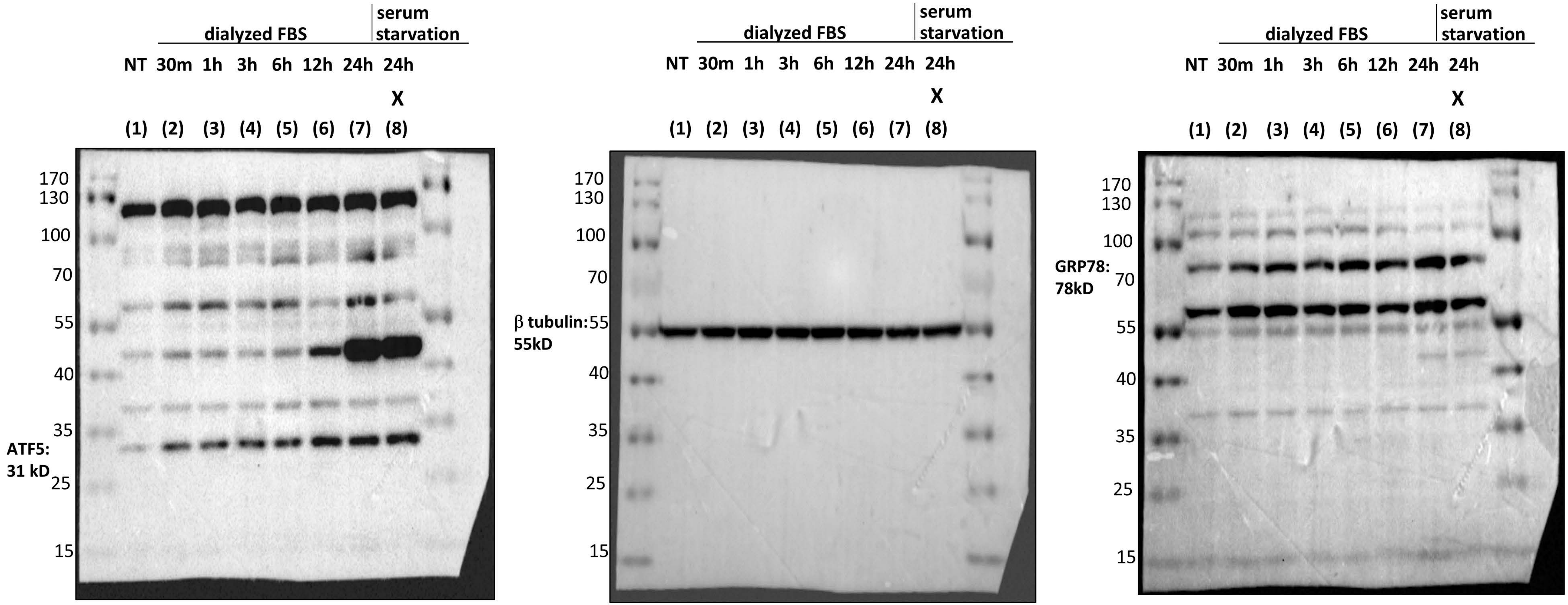

HeLa cells were transfected with vector control (GFP) and treated as described. NT= not treated. Blots were probed for ATF5, molecular weight ~31kD; β tubulin, molecular weight ~55kD, and GRP78, molecular weight ~78kD. Images were captured using the Bio-Rad Chemidoc XRS, after incubation with horseradish peroxidase-coupled species-specific secondary antibodies and ECL Western blot detection reagent.

Uncut western blot images from Figure 4.

Panel D

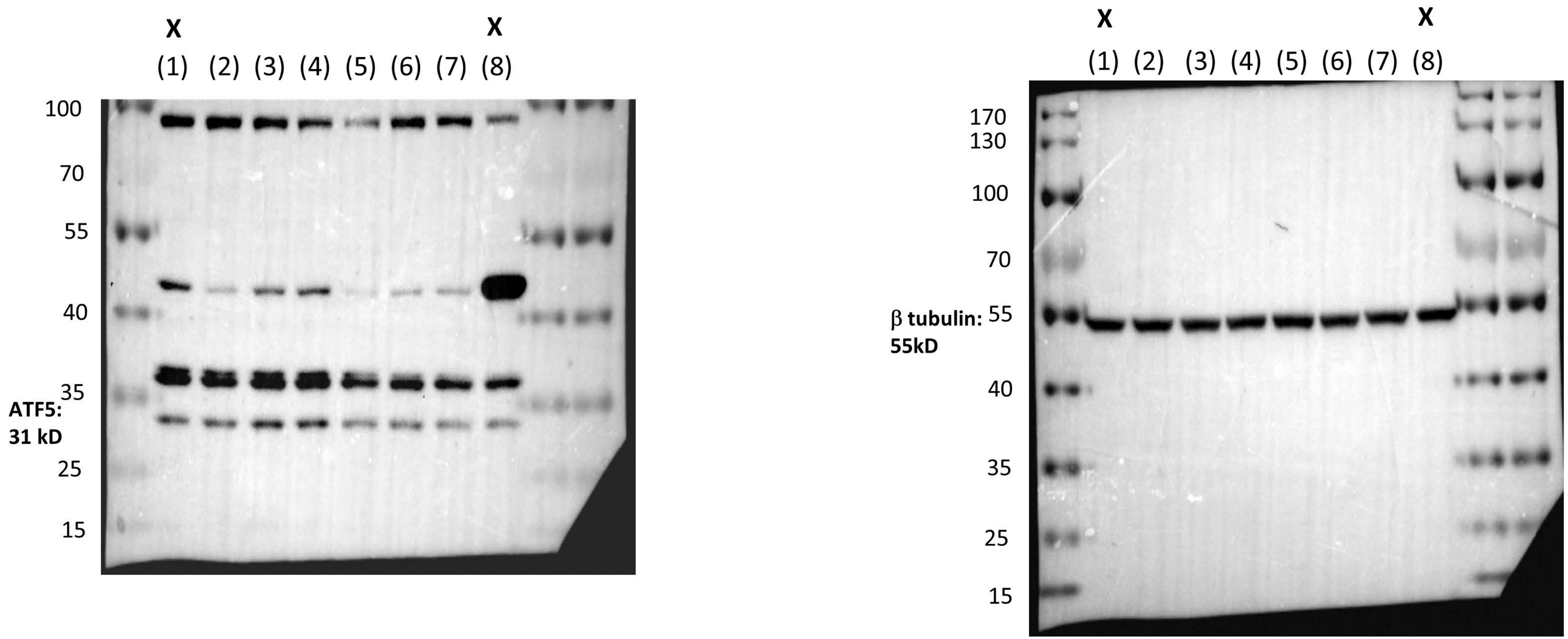

HeLa cells were transfected treated with dialyzed FBS for 24h as described. Lanes 1: neg. control, lane 2: neg. control, lane 3: pre-miR-129, lane 4:, pre-miR-433-3p, lane 5: pre-miR-520b, lane 6: all three pre-miRNA co-transfected, lane 7: pos. control, lane 8: neg. control + serum starvation (24h). ATF5, molecular weight ~31kD was probed, as was β tubulin, molecular weight ~55kD. Images were captured using the Bio-Rad Chemidoc XRS, after incubation with horseradish peroxidase-coupled species-specific secondary antibodies and ECL Western blot detection reagent.

Uncut western blot images from Figure 6.

Panel A

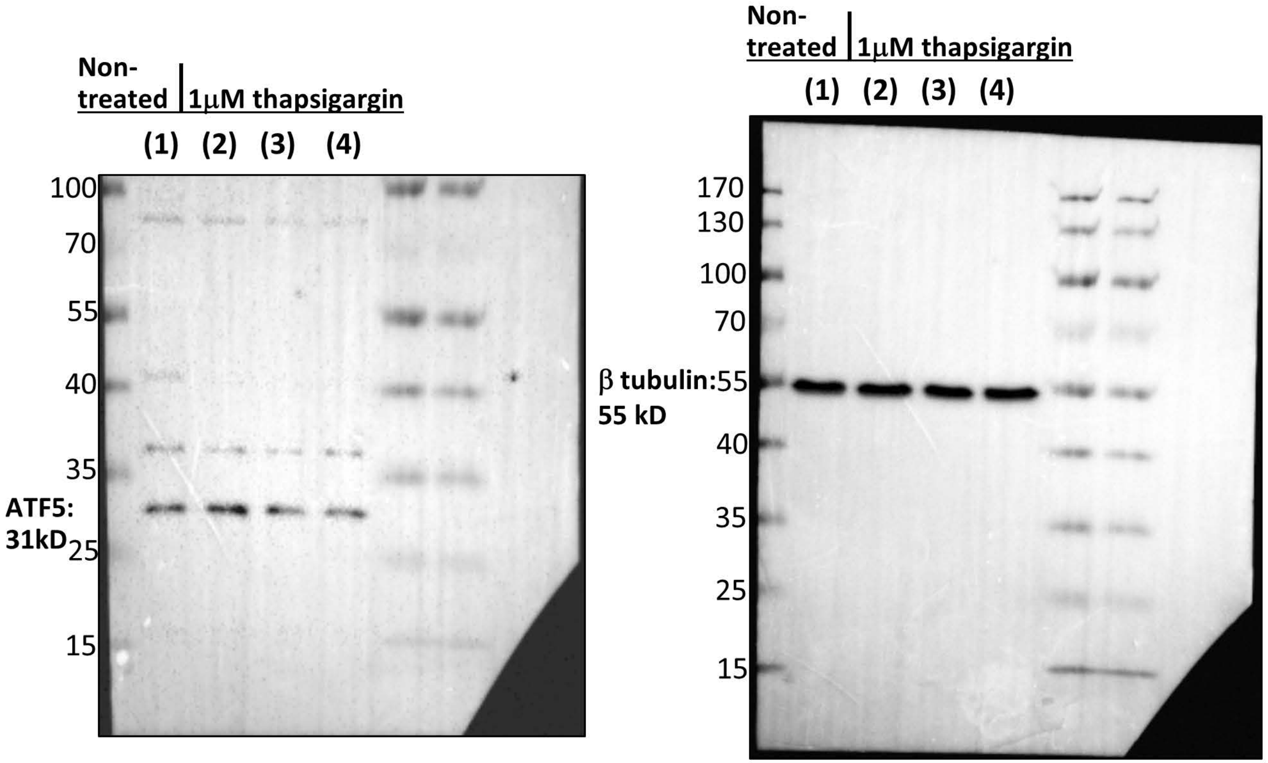

Panel B

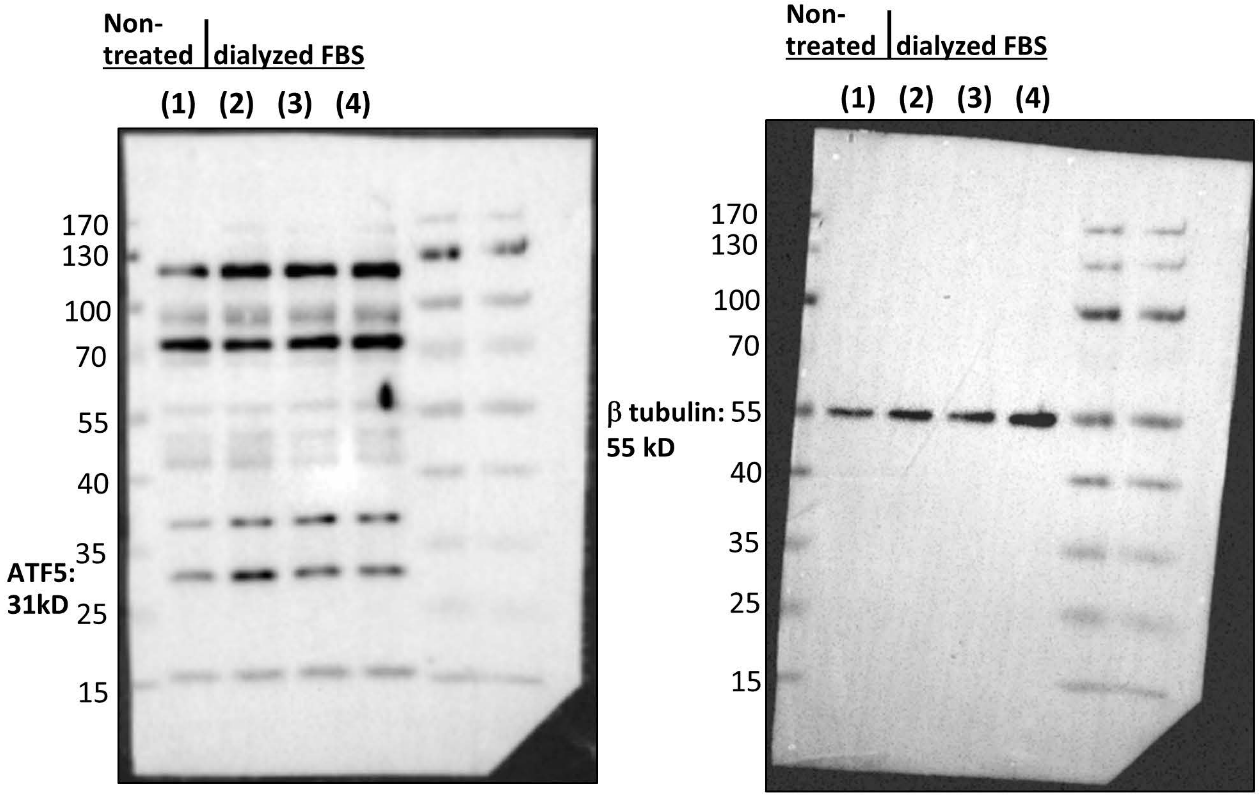

MCF-7 cells were transfected and treated as described. Lanes 1-2: negative control, lane 3: pre-miR-520b, lane 4: positive control. Blots were probed for ATF5, molecular weight ~31kD and β tubulin, molecular weight ~55kD. Images were captured using the Bio-Rad Chemidoc XRS, after incubation with horseradish peroxidase-coupled species-specific secondary antibodies and ECL Western blot detection reagent.
